# *Christensenella* regulated by Huang-Qi-Ling-Hua-San is a key factor by which to improve type 2 diabetes

**DOI:** 10.3389/fmicb.2022.1022403

**Published:** 2022-10-12

**Authors:** Tong Pan, Shujun Zheng, Weiwei Zheng, Chao Shi, Ke Ning, Qinghui Zhang, Yanbo Xie, Hongyu Xiang, Qiuhong Xie

**Affiliations:** ^1^National Engineering Laboratory for AIDS Vaccine, School of Life Sciences, Jilin University, Changchun, China; ^2^School of Life Sciences, Jilin University, Changchun, China; ^3^Jilin Provincial Key Laboratory of Agricultural Biotechnology, Jilin Academy of Agricultural Sciences, Changchun, China; ^4^Key Laboratory for Molecular Enzymology and Engineering of Ministry of Education, School of Life Sciences, Jilin University, Changchun, China; ^5^Institute of Changbai Mountain Resource and Health, Jilin University, Fusong, China

**Keywords:** traditional Chinese medicine, gut microbiota, type 2 diabetes, FMT, *Christensenella*, metabolomics

## Abstract

There is a lot of evidence that oral hypoglycemic drugs work by affecting gut microbes, but the key strains responsible for this effect are not well known. Huang-Qi-Ling-Hua-San (HQLHS), composed of *Astragalus Membranaceus*, *Ganoderma lucidum*, *Inonotus obliquus*, and *Momordica charantia* L., is a specially designed Chinese medicine formula to treat type 2 diabetes (T2D). In this study, a mouse model of T2D induced by high-fat diet and streptozotocin was used to explore the mechanism of HQLHS in improving hyperglycemia and hyperlipidemia through multiple rounds of animal experiments, such as HQLHS feeding, fecal microbiota transplantation (FMT), and live bacteria feeding, so as to explore the potential target intestinal flora in its hypoglycemic effect. Results show that such specific taxa as *Bifidobacterium*, *Turicibacter*, *Alistipes*, *Romboutsia*, and *Christensenella* were identified to be preferably enriched by HQLHS and then assumed to be the target microbes. Herein, FMT was used to test if the upregulated beneficial bacteria by HQLHS play a therapeutic role. The strain *Christensenella minuta* DSM 22607 and the strain *Christensenella timonensis* DSM 102800 were selected to test the beneficial effect of *Christensenella* taxa on T2D. Diabetic animals supplemented with these strains showed the improvement in blood glucose and lipid metabolism, the promotion of GLP-1 secretion, the increase in antioxidant capacity, the inhibition of hepatic gluconeogenesis, the suppression of intestinal glucose absorption, the enhancement of intestinal barrier, reduced LPS-induced inflammation, and the reduction of branched amino acids (BCAAs) content in the liver. Overall, these data demonstrate that *Christensenella* plays a beneficial role in T2D and is a target for the action of HQLHS therapy.

## Introduction

Type 2 Diabetes (T2D) is a complex endocrine and metabolic disease characterized by hyperglycemia. T2D causes varying degrees of insulin resistance, pancreatic β cell dysfunction, low-grade systemic inflammation, intestinal flora imbalance, and other endocrine disorders under the interaction of genetic and environmental factors (Stumvoll et al., 2005; Kahn et al., 2006; Tahrani et al., 2016). The global trend of T2D poses a major challenge to the health care systems, and in 2040, the prevalence is predicted to be 13.5% worldwide (Saeedia et al., 2019).

Recently, studies on the pathogenesis and therapeutic targets of T2D have focused on the structure of the intestinal microflora in humans and animal models (Sanna et al., 2019). Manipulation of the gut microbiota of the gut microbiota, such as antibiotic administration, probiotics addition, and fecal microbiota transplantation (FMT), may be useful for T2D therapy (Leylabadlo et al., 2020).

Traditional Chinese medicine (TCM) is an important form of complementary medicine that has become an attractive strategy in the treatment of diabetes (Wang et al., 2019). Since it mainly depends on oral administration, TCM directly impacts the intestinal flora. However, most active ingredients derived from herbal medicines, such as triterpene glycosides, flavonoids, and tannins, exhibit properties that limit TCM bioavailability, e.g., poor lipophilicity, high hydrogen bonding capacity, and high molecular flexibility. The biotransformation by intestinal flora facilitates the absorption, which therefore plays an important pharmacological role (Xu et al., 2017; Nie et al., 2018). Also, TCM regulates the community of host intestinal microflora since many TCM components are nutrients for the growth of specific bacteria (Feng et al., 2019).

Numerous studies have confirmed that TCM alters specific intestinal bacteria during T2D treatment and its complications. For example, Liu et al. (2019) reported that polysaccharide in *Astragalus membranaceus* improves cognitive dysfunction in diabetic mice by altering gut microbiota, such as dramatically enriching the abundance of *Lactobacillus* and *Sutterella* in the gut. A polysaccharide in *Ganoderma lucidum* improved to be effective in improving glucose and lipid metabolism, reducing inflammation, and increasing the relative abundance of short-chain fatty acid (SCFA)-producers (Chen et al., 2020). In diabetic rats, fermented *Momordica charantia* L. juice mitigated the hyperglycemia, hyperinsulinemia, hyperlipidemia, and oxidative stress and this was associated with an increase in *Bacteroides*, *Oscillibacter*, and *Prevotella* (Gao et al., 2019). Moreover, formula BuZangTongLuo decoction prevented diabetic complications by increasing the abundances in *Akkermansia*, *Bifidobacterium*, and *Bacteroides*, while decreasing the populations of *Blautia*, *Weissella*, and *Kurthia* (Zheng et al., 2020). Overall, exploring the changes of gut microbiota is crucial for understanding the pharmacological mechanisms of TCM for treating T2D.

Among many observations, the genera like *Bifidobacterium*, *Lactobacillus*, *Faecalibacterium*, *Akkermansia*, and *Roseburia* are consistent to be negative correlation with T2D characteristics after oral Chinese medicine intervention. And these bacteria are commonly used as probiotics by different protection mechanisms (Gurunga et al., 2020). For instance, *Lactobacillus rhamnosus* GG (LGG) interacts with host cells through Metabolism-Associated Molecular Patterns (MAMPs) to modulate the immune response (Li Y B, et al., 2020). Both *Akkermansia muciniphila* and pasteurised *A*. *muciniphila* are able to reduce lipid accumulation and improve gut barrier (Depommier et al., 2020; Lee et al., 2022). *Christensenellaceae*, a Gram-negative human gut commensal bacterium belonging to phylum *Firmicutes*, is emerging as a vital player in human health (Waters and Ley, 2019). Guillaume et al. (Déjean et al., 2021) reported that *Christensenella minuta* carries a bile salt hydrolase (BSH) protein, which may regulate bile acid circulation in the host. Many studies have found that *Christensenellaceae* are negatively correlated with the pathologies typical of metabolic syndrome, however, the underlying mechanism has not been fully explained. Thus, Christensenellaceae strains as the next generation of probiotics being developed, and it is necessary to explain how they play a beneficial role in the intestinal ecosystem (Li X, et al., 2020).

Although it is well known that TCM therapy modifies intestinal microbiota, its effect on enriched key bacteria has rarely been explored. The research aimed to identify the key bacterial communities that respond to HQLHS in glycemic control. FMT confirmed the key role of the intestinal beneficial bacteria enriched by the Chinese medicine formula. The subsequent specific strains intervention suggests that *Christensenella* is the target gut bacteria taxa in the hypoglycemic process of HQLHS, and the potential mechanism was explored.

## Materials and methods

### Preparation of the Chinese medicinal formula Huang-Qi-Ling-Hua-San

For the preparation of HQLHS: *Astragalus Membranaceus*, *Ganoderma lucidum*, *Inonotus obliquus*, and *Momordica charantia* L. were provided by Jilin Tongren Pharmacy (Changchun, China). The four kinds of medicinal materials were pulverized and screened, respectively, and mixed at a ratio of 2:1:1:1.

The detection of the main components was entrusted to Beijing Novogene Co. Ltd., performing by high-performance liquid chromatography-triple quadrupole linear ion trap tandem mass spectrometry (HPLC-QTrap-MS/MS) based on the highly sensitive SCIEX QTRAP® 6500+ mass spectrometry platform. Quantitative data acquisition was accomplished in multiple reaction monitoring (MRM) mode, and further identification of target compounds was achieved by ultra-performance liquid chromatography-quadrupole-time-of-flight mass spectrometry. HPLC parameter: the Waters XselectTM HSS T3, 2.5 μm, 2.1 mm × 150 mm column, mobile phase A 0.1% formic acid, mobile phase B 0.1% formic acid-acetonitrile, 50°C, 0.4 ml/min gradient elution.

### Strains and medium

*Christensenella minuta* DSM 22607 and *Christensenella timonensis* DSM 102800 were purchased from the German Collection of Microorganisms and Cells Cultures (DSMZ). Both strains were cultured in Reinforced Clostridial Medium (BD Difco/BBL) under anaerobic conditions at 37°C and obtained by centrifugation (10,000 r/min, 1 min) after 4 days. After washing three times with normal saline, the solution was diluted into 1 × 10^9^ cfu/ml for intragastric administration.

### Animal experiments

Specific pathogen-free (SPF) male C57BL/6 J mice (6–8 weeks old, 18–22 g) were purchased from Vital River Laboratory Animal Technology Co., Ltd. (Beijing, China). Normal diet feed (TP 23402) and 60% high-fat feed (TP 23400) were purchased from TROPHIC Animal Feed High-tech Co., Ltd. All animals were maintained in accordance with the laboratory animal guidelines, and the animal experiments were approved by the Institutional Animal Care and Use Committee of Jilin University (Permission Number: 2018-SY1005).

#### Part I

After 1 week of acclimation, the animals were randomly divided into four groups (*n* = 10). For the remaining 13 weeks, 10 mice in the normal control (NC) group were fed with normal diet and the other 30 mice were fed with 60% high-fat diet. The 30 high-fat diet mice were intraperitoneally injected with STZ (dissolved in 120 mg/kg in ice cold citrate buffer, pH = 4.3) by the fourth week. Meanwhile, NC group was injected with citrate buffer. One week after the STZ injection, tail blood was collected to measure fasting blood glucose (FBG). Mice with fasting blood glucose >11.1 mmol/L were considered to have T2D. After the fifth week, the diabetic mice were treated by gavage once a day with normal saline (DM), 300 mg/kg/metformin dissolved in normal saline (MET) and 800 mg/kg HQLHS dissolved in normal saline (DM-HQ).

#### Part II

After 1 week of acclimation, the animals were randomly divided into five groups (*n* = 10). The NC group received normal saline and the NC-HQ group received 800 mg/kg/day HQLHS dissolved in normal saline. Both groups were fed with a normal diet. The remaining 30 mice were kept on a 60% high-fat diet and, as in Part I, after the fourth week they were intraperitoneally injected with 120 mg/kg STZ. After the fifth week, T2D mice (FBD > 11.1 mmol/L) were treated by gavage once a day normal saline daily (DM), 300 μl bacterial suspension (FMT), and 800 mg/kg HQLHS dissolved in normal saline (DM-HQ) by gavage once a day. At the fifth week, fecal samples were obtained from the NC-HQ group every day for FMT. Samples were homogenized and suspended in the normal saline (500 mg in 5 ml) followed by centrifugation at 800 rpm for 3 min. Each FMT group mouse was daily gavaged 300 μl bacterial suspension.

#### Part III

After 1 week of acclimation, all the animals were fed with 60% high-fat diet throughout the experiment. The modeling method of T2D induced by STZ is described in Part I. After the fifth week, T2D mice were treated by gavage once a day with 300 μl of normal saline (DM), *Christensenella minuta* DSM 22607 (1 × 10^9^ cfu/ml) suspension (CM), and *Christensenella timonensis* DSM 102800 (1 × 10^9^ cfu/ml) suspension (CT), where each strain was suspended in normal saline.

The oral glucose tolerance test (OGTT) was measured at week 11. After 12 h of fasting, mice were orally administered with 2.0 g/kg glucose, and tail vein blood samples were collected for testing after 0, 30, 60, 90, and 120 min. Blood glucose levels were measured using a SUNNUO glucometer (Sinocare Inc., Changsha, China).

### Sample collection

After the last administration, fresh feces were collected and preserved at −80°C for 16S rDNA sequencing. Blood was collected *via* ocular sampling after the experiment and the serum was obtained by centrifugation of blood samples at 3,000 × g for 15 min at 4°C. The epididymis fat, liver, pancreas, ileum, colon, and cecal contents were collected after dissecting the mice. Part of the liver, colon, and pancreas were preserved in 4% paraformaldehyde for morphological observation by H&E staining. The remaining tissues and the cecal contents were stored at −80°C for further study.

### Serum and liver biochemical analysis

Biochemical analyses of serum and liver samples included liver glycogen, aspartate aminotransferase (AST), alanine aminotransferase (ALT), superoxide dismutase (SOD), glutathione peroxidase (GSH-Px), catalase activity (CAT), malondialdehyde (MDA), total cholesterol (T-CHO), triglyceride (TG), low-density lipoprotein cholesterol (LDL-C), and high-density lipoprotein cholesterol (HDL-C) using commercially available assay kits (Nanjing Jiancheng Bioengineering Institute, China). Serum Insulin (INS), glucagon-like peptide-1 (GLP-1), and lipopolysaccharide (LPS) were detected using the enzyme-linked immunosorbent assay (ELISA) kit (Cusabio Biotechnology, Wuhan, China) according to the manufacturer’s instruction.

### RNA extraction and quantitative real-time PCR

The total RNA extraction, RT-PCR, and qPCR process were performed as described (Wang et al., 2020). The primer sequences are shown in Supplementary Table 2, and 18S rRNA was used as the reference gene.

### Gut microbiota analysis of cecal and fecal content

Samples (cecal contents) of mice belonging to the same cage were mixed and treated as one sample (according to the coprophagic character of the mice) for testing. Three samples were obtained from each group of mice. Bacterial DNA was extracted from the cecal contents for 16S rDNA gene sequencing analysis. Detailed procedures of 16S rDNA gene V3–V4 region sequencing and data processing were followed a previous report (Xiang et al., 2022). The V3–V4 region of the 16S rRNA gene was amplified with 338F (5′-CCTACGGRRBGCASCAGKVRVGAAT-3′) and 806R (5′-GGACTACNVGGGTWTCTAATCC-3′). High-throughput sequencing and analysis of bacterial rDNA genes were performed at Beijing Biomarker Technologies Co., Ltd. using the IlluminaHiseq 2500 platform. The second generation data processing flow was as follows: Trimmomatic (version 0.33) was used to filter the quality of the raw data; Cutadapt (version 1.9.1) was used to identify and remove the primer sequences; USEARCHUSEARCH (Version 10) was used to splice the double-ended reads and remove chimeras (UCHIME, version 8.1). Finally, high-quality sequences were obtained for subsequent analysis.

### NMR metabolomics analysis and sample processing

NMR data acquisition and processing were performed as described (Yu et al., 2020). Data collection was performed on the public instrument platform of Jilin University. All samples were analyzed by an AVANCE III 600 M MHz NMR spectrometer at 298.2 K. 1D ^1^H NMR spectra were acquired using CPMG, with relaxation delay of 3 s and a mixing time of 0.1 s. Sixty-four free induction decays (FIDs) were collected into 64 K data points and an acquisition time of 2 s. FIDs were zero-filled to 64 K prior to Fourier transformation.

Sample preparation was performed as described (Mora-Ortiz et al., 2019), with some modifications: Briefly, the liver tissues of three mice from each cage were thawed and at room temperature. A total of 150 mg of liver samples (about 50 mg from each mouse) was taken, suspended with 1.8 ml pre-cooled methanol–water (2/1, v/v), and lysed with magnetic beads. After centrifugation for 10 min (8,000 × *g*, 4°C), the supernatant of each sample was collected. The above process was repeated twice. The supernatant of each sample was combined and centrifuged again for 10 min. After methanol was removed under vacuum, the supernatants of each sample were freeze-dried, producing hydrophilic extracts of liver. The samples obtained were hydrophilic extracts of liver. The remaining solid residue of each tissue sample was transferred to a new EP tube, extracted with 1.8 ml precooled chloroform, and the mixture was treated with five intermittent ultrasound treatments (60 s ultrasound/60 s crushing). After centrifugation (8,000 × *g*, 4°C), the chloroform layer was collected carefully. The process was repeated twice, and the combined layer was centrifuged again for 10 min. Chloroform was removed from the sample supernatant under vacuum to obtain the lipophilic extract of the liver.

NMR raw data were preprocessed by MestReNova 10.0. The statistical strategy adopted for the analysis of the samples involved a preliminary unsupervised Principal Component Analysis (PCA), followed by a supervised pairwise Orthogonal Projection to Latent Structures Discriminant Analysis (O2PLS-DA) using SIMCA 14.0 (Umetrics, Umeå, Sweden). To validate the models, random permutation testing (200 randomizations) was then applied. The Variable Importance in the Projection (VIP) value (VIP > 1, *p* < 0.05) was used to evaluate the variable contribution and to identify the potential biomarkers. MetaboAnalyst was applied to screen out important metabolic pathways related to differential metabolites, which were obtained by KEGG.^1^ Metabolite identification was done using publicly available online databases: the Human Metabolome Data Base (HMDB)^2^, based on chemical shifts of hydrogen and peak multiplicity (Supplementary Table 3).

### Statistical analysis

All data are presented as the mean values ±SEM. Statistical differences and Spearman’s correlation were determined by ANOVA analysis and Tukey’s test. Data were analyzed using SPSS 24.0 (SPSS Inc., Chicago, IL, United States) and performed with Origin 2018 (Origin Lab, Northampton, MA, United States). Visual network diagram was performed on Cytoscape_3.9.0. The area of the pancreatic islets of Langerhans was calculated by Auto CAD 2021. Hierarchical clustering tree and Principal co-ordinates analysis (PCoA) plots were generated by the ggplot2 packages of the R software (version 3.3.1). Differentially abundance of genera across groups was calculated using an extended error bar plot using STAMP (Ver. 2.1.3). LEfSe analysis was performed on http://huttenhower.sph.harvard.edu/galaxy/. Significant differences were assumed for values of *p* < 0.05.

## Results

### HQLHS intervention regulates gut microbiota during the reversal of T2D

The determination of the main components of Chinese medicine prescription is to expound its pharmacodynamic substance basis in this study. In HQLHS, 1596 components were detected by LC–MS (Supplementary Table 1), consisting mainly of flavonoids, aliphatic acrylates, amino acids, and derivatives. Among these, calytoisoflavone and tricytoisoflavone accounted for 10% of the total.

**Figure 1:**
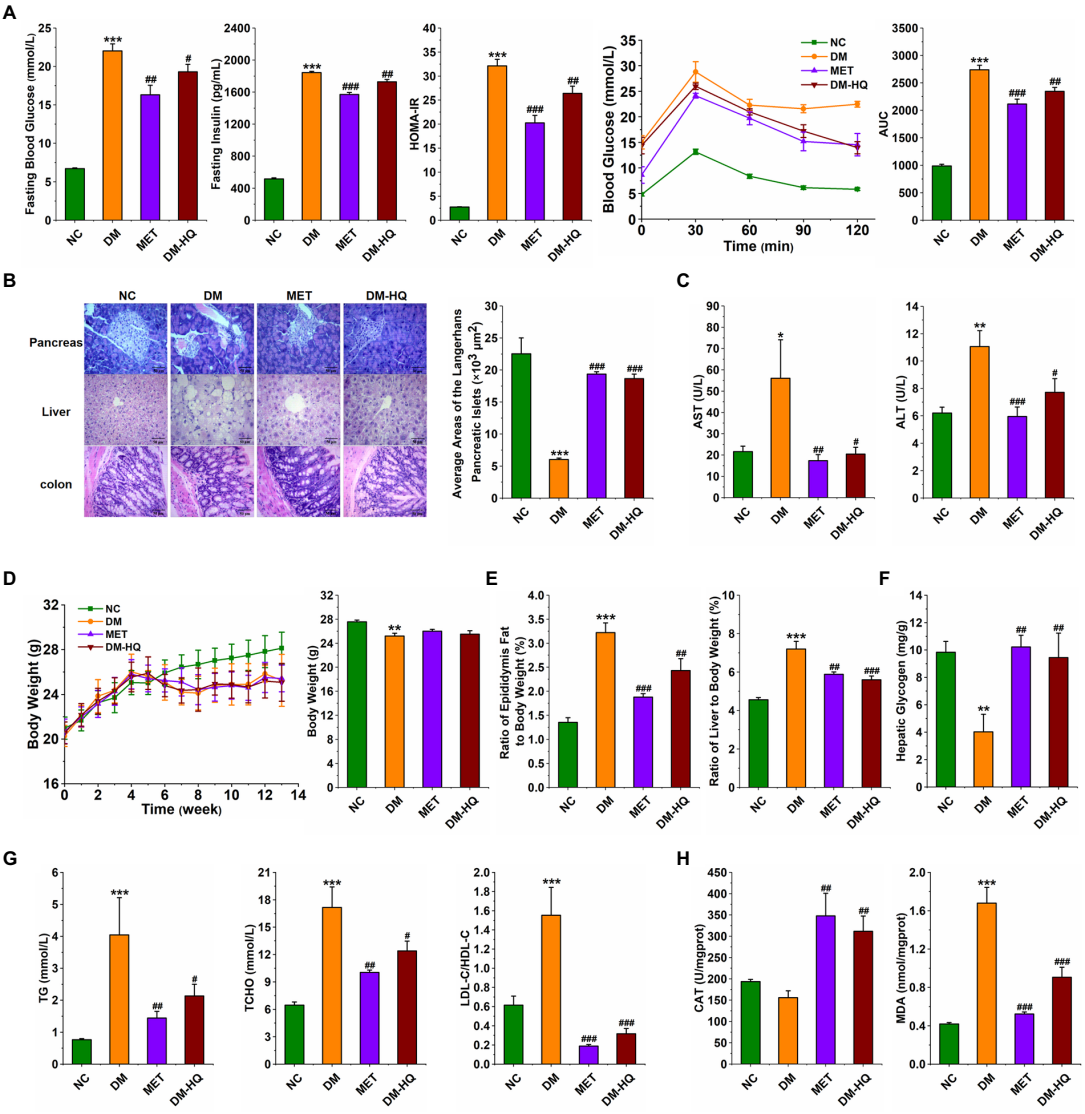
Huang-Qi-Ling-Hua-San (HQLHS) attenuated HFD and STZ induced type 2 diabetes. **(A)** Fasting blood glucose, fasting insulin level, homeostasis model assessment-insulin resistance (HOMA-IR), oral glucose tolerance test (OGTT), and area under the curve (AUC). **(B)** Morphological observation of pancreas (magnification, ×200, 50 μm), liver (magnification, ×200, 50 μm), colon (magnification, ×400, 50 μm) with H&E staining, and the average area of the pancreatic islets of Langerhans. **(C)** Serum levels of aspartate aminotransferase (AST) and alanine aminotransferase (ALT). **(D)** The weekly weight change and the final weight at 13th week in the four groups. **(E)** Epididymal fat index and liver index. **(F)** Level of hepatic glycogen. **(G)** Blood triglycerides, cholesterol, LDL-C/HDL-C levels. **(H)** Serum levels of Catalase (CAT) and malondialdehyde (MDA). Data are expressed as mean ± SEM; One-way ANOVA was used to analyze statistical differences; Compare to NC: *p < 0.05; **p < 0.01; ***p < 0.001; Compare to DM: #*p* < 0.05; ##*p* < 0.01; ###*p* < 0.001.

In order to explore the anti-diabetes effect of HQLHS, the part I animal experiment was designed (Supplementary Figure 1), where the blood glucose, blood lipid, and intestinal microflora in STZ-induced diabetic mice were analyzed and compared with metformin. HQLHS significantly decreased the levels of fasting blood glucose, fasting insulin, and homeostasis model assessment-insulin resistance (HOMR-IR; Figure 1A) and increased liver glycogen content (Figure 1F). The DM-HQ group showed better glucose clearance during the oral glucose tolerance test (OGTT; Figure 1A). STZ and HFD treatment resulted in severe enlargement of liver cell volume, a reduction in the size of Langerhans islet cells, incomplete colonic mucosal barrier, and inflammatory cell infiltration, whereas HQLHS treatment reversed this situation to some extent (Figure 1B). Compared to the DM group, DM-HQ mice presented lower serum concentrations of alanine transaminase (ALT) and aspartate aminotransferase (AST; Figure 1C). HQLHS-treated mice showed improved features of lipid metabolism, characterized by lower the serum level of triglycerides (TG), lower the total cholesterol (T-CHO), and reduced ratio between low and high-density lipoprotein cholesterol (LDL-C/HDL-C; Figure 1G). Additionally, there was a reduced liver and epididymis fat index, but no effect on body weight (Figures 1D,E). Some antioxidant supplements can be used as an adjunct therapy for diabetes, reducing oxidative stress and helping to slow or prevent complications (Al-Aubaidy and Jelinek, 2011; Mirmiranpour et al., 2013). HQLHS increased the activity of serum catalase (CAT) activity and decreased serum malondialdehyde (MDA) content (Figure 1H), indicating that it has antioxidant properties.

To investigate the effect of HQLHS on gut microbiota, we analyzed the cecal content flora of the four groups of mice. HQLHS supplementation did not significantly change the overall gut microbiota abundance in the diabetic mice, as shown by α diversity (Table 1), whereas the ACE index was significantly different in MET compared to the DM group.

**Table 1:** Effects of HQLHS on α diversity of cecal microbiota in diabetic mice.

	ACE	Chao1	Simpson	Shannon
NC	240 ± 6.12^b^	274 ± 9.77^b^	0.22 ± 0.05^a^ ^b^	2.08 ± 0.22^b^
DM	280 ± 6.05^a^	279 ± 4.48^a^ ^b^	0.14 ± 0.04^a^ ^b^	3.06 ± 0.23^a^ ^b^
MET	241 ± 13.7^b^	237 ± 17.0^b^	0.24 ± 0.05^a^	2.16 ± 0.26^b^
DM-HQ	291 ± 3.80^a^	294 ± 4.28^a^	0.06 ± 0.00^b^	3.77 ± 0.04^a^

Data were expressed as mean ± SEM.

a Means with different superscripts within the same row differ based on Tukey’s test (*p* < 0.05).

b Means with different superscripts within the same row differ based on Tukey’s test (*p* < 0.05).

**Figure 2:**
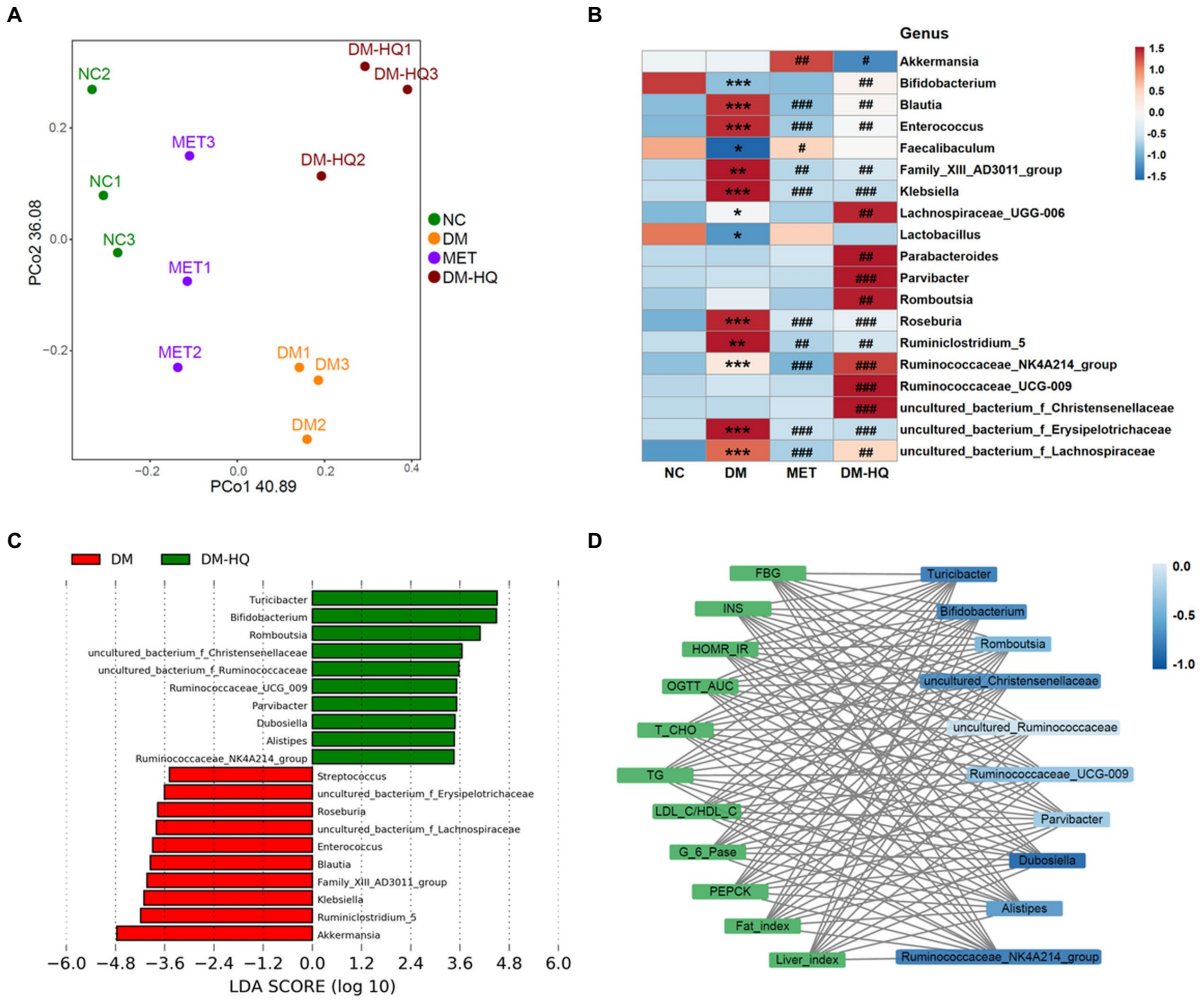
HQLHS regulated the intestinal flora disorder caused by type 2 diabetes. **(A)** Principal co-ordinates analysis (PCoA) of the cecal content microbiota. **(B)** Heat map statistics of significant difference genus. **(C)** Analysis of differences in the microbial taxa shown by [linear discriminant analysis (LDA; LEfSe) coupled with effect size measurements] between the DM group and the DM-HQ group (log10 LDA > 2.0). **(D)** Visualization of the correlation network between glycolipid parameters and dominant genus in the DM-HQ group based on Spearman’s rank correlation coefficient. Data are expressed as mean ± SEM. One-way ANOVA was used to analyze statistical differences; Compare to NC: *p < 0.05; **p < 0.01; and ***p < 0.001; Compare to DM: ^#^*p* < 0.05; ^##^*p* < 0.01; and ^###^*p* < 0.001.

However, based on the PCoA scores, there was a clear separation between the DM group and NC, MET, and DM-HQ groups (Figure 2A). At the phylum level, *Bacteroidetes*, *Firmicutes*, and *Proteobacteria* were predominant phyla in the cecal flora (Supplementary Figure 2 A). The relative abundance of *Bacteroides* in the DM-HQ group was higher than that in the DM group, while the abundance of *Firmicutes* did not significantly change (Supplementary Figure 2 B). From the heatmap, the MET group significantly reduced the relative abundance of certain bacteria enriched by diabetes, such as *Blautia*, *Klebsiella*, *Roseburia*, *Ruminiclostridium_5*, *uncultured_bacterium_f_Erysipelotrichaceae* and *Family_XIII_AD3011_group*. In line with previous reports (Liu et al., 2018; Zhang and Hu, 2020), metformin treatment significantly raised the relative abundance of *Akkermansia* and *Faecalibaculum* (Figure 2B). Beyond this, compared with diabetic mice, the DM-HQ group significantly increased the relative abundance of *Bifidobacterium*, *Romboutsia*, *Parabacteroides*, *Parvibacter*, *Ruminococcaceae_UCG-009*, *Lachnospiraceae_UGG-006*, *Ruminococcaceae_NK4A214_group*, *uncultured_bacterium_f_Ruminococcaceae*, *uncultured_bacterium_f_Lachnospiraceae* and *uncultured_bacterium_f_Christensenellaceae*, while significantly reducing the relative abundance of *Akkermansia*, *Blautia*, *Klebsiella*, *Roseburia*, Ruminiclostridium_5, *uncultured_bacterium_f_Erysipelotrichaceae*, and *Family_XIII_AD3011_group* (Figure 2B). Furthermore, the LDA score ulteriorly indicates the degree of influence of statistical differences among different species. *Turicibacter*, *Bifidobacterium*, *Romboustia*, *Parvibacter*, *Alistipes*, *Ruminococcaceae_NK4A214_group*, *Ruminococcaceae_UCG_009*, *Dubosiella*, *uncultured_bacterium_f_Ruminococcaceae*, and *uncultured_bacterium_f_Christensenellaceae* were the dominant taxa of the DM-HQ group and could be regarded as the potential biomarker (Figure 2C).

Correlation analysis showed that the improvement of hyperglycemia and hyperlipidemia was associated with changes in intestinal flora (Supplementary Figure 3). *Family_XIII_AD3011_group* was significantly positively correlated with FBG, HOMA-IR, AST, TG, and MDA. Significant positive correlations were observed between *Klebsiella* and OGTT, LDL-C, fat index, and MDA. *Enterococcus* was positively correlated with fat index. Conversely, *Turicibacter*, *Alistipes*, *Bifidobacterium*, *Romboutsia*, *Ruminococcaceae_NK4A214_group*, *Parabacteroides*, *Dubosiella*, *Ruminococcaceae_UCG-009*, *uncultured_bacterium_f_Ruminococcaceae*, and *uncultured_bacterium_f_Christensenellaceae* were negatively correlated with the parameters associated to abnormal blood glucose and lipid metabolism. For a clearer display, a visual network diagram (Figure 2D) was applied for analysis. Obviously, *Turicibacter*, *Bifidobacterium*, and *uncultured_bacterium_f_Christensenellaceae* showed a strong negative correlation with glycolipid parameters. These results suggest that the Chinese medicine formula might alleviate T2D by altering the gut flora.

### HQLHS was able to stably regulate specific intestinal bacteria in mice

In order to identify the specific intestinal bacteria responding to HQLHS treatment, we treated NC mice with HQLHS (NC-HQ group) and collected the feces from NC-HQ mice at week 0 [NC-HQ(0)] and at week 4 (NC-HQ; Saeedia et al., 2019) for 16S rDNA sequencing.

**Figure 3:**
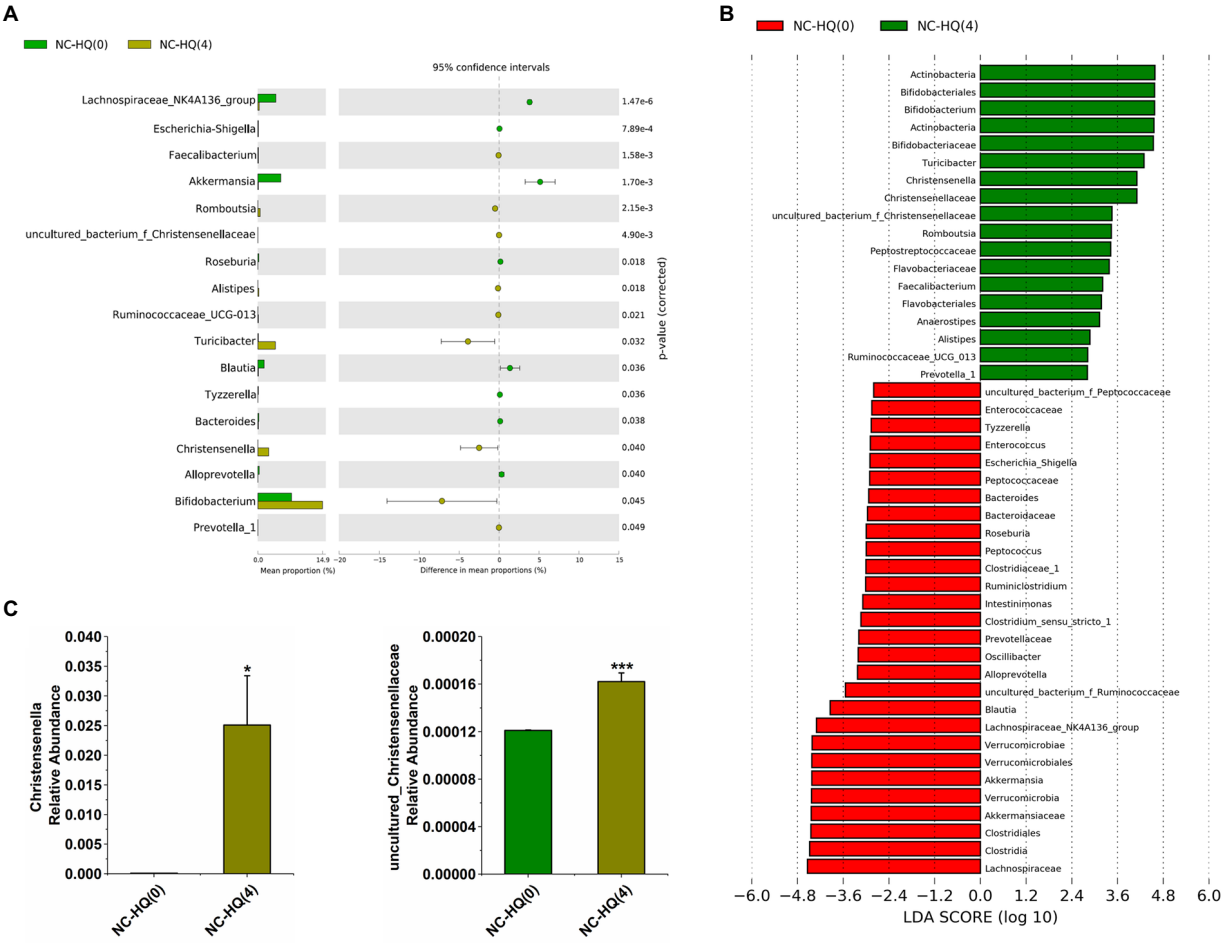
**(A)** Differential abundant features of genus before and after HQLHS treatment. **(B)** Discriminative taxa determined by LEfSe between the two groups (log_10_ LDA > 2.0). **(C)** Comparison proportion of *Christensenella* and uncultured_bacterium_f_Christensenellaceae in feces before and after HQLHS treatment. Statistical analysis was performed using Student’s *t*-test between NC-HQ(0) and NC-HQ (Saeedia et al., 2019) groups. Data are expressed as mean ± SEM. Compare to NC-HQ(0): *p < 0.05; ***p < 0.001.

After 4 weeks of HQLHS administration, similar to the previous results, it can be seen from the extended error bar plot (Figures 3A,C) shows that *Turicibacter*, *Alistipes*, *Bifidobacterium*, *Romboutsia*, *Christensenella*, *uncultured_bacterium_f_Christensenellaceae*, *Ruminococcaceae_UCG-013*, and *Prevotella_1* were significantly upregulated, while *Akkermansia*, *Roseburia*, and *Blautia* were significantly downregulated. In contrast, the relative abundance of *Faecalibacterium* was significantly increased, while *Ruminococcaceae_NK4A214_group* was significantly reduced. The differences in the intestinal flora may be due to different diets in the NC and DM groups, and to individual differences in the different batches of mice may lead to different intervention effects of HQLHS on intestinal flora. In addition, the relative abundance of *Escherichia-Shigella*, *Tyzzerella*, *Bacteroides*, and *Alloprevotella* was significantly decreased. After HQLHS intervention, according to the linear discriminant analysis (LDA) value (Figure 3B), *Turicibacter*, *Bifidobacterium*, *Romboutsia*, *Christensenella*, and *uncultured_bacterium_f_Christensenellaceae* displayed significant differences.

Combined with the Part I work, these results show that HQLHS intervention consistently increased *Turicibacter*, *Alistipes*, *Bifidobacterium*, *Romboutsia*, *Christensenella*, and certain taxa of *Ruminococcaceae*.

### Transplantation of gut microbiota altered by HQLHS alleviated T2D

**Figure 4:**
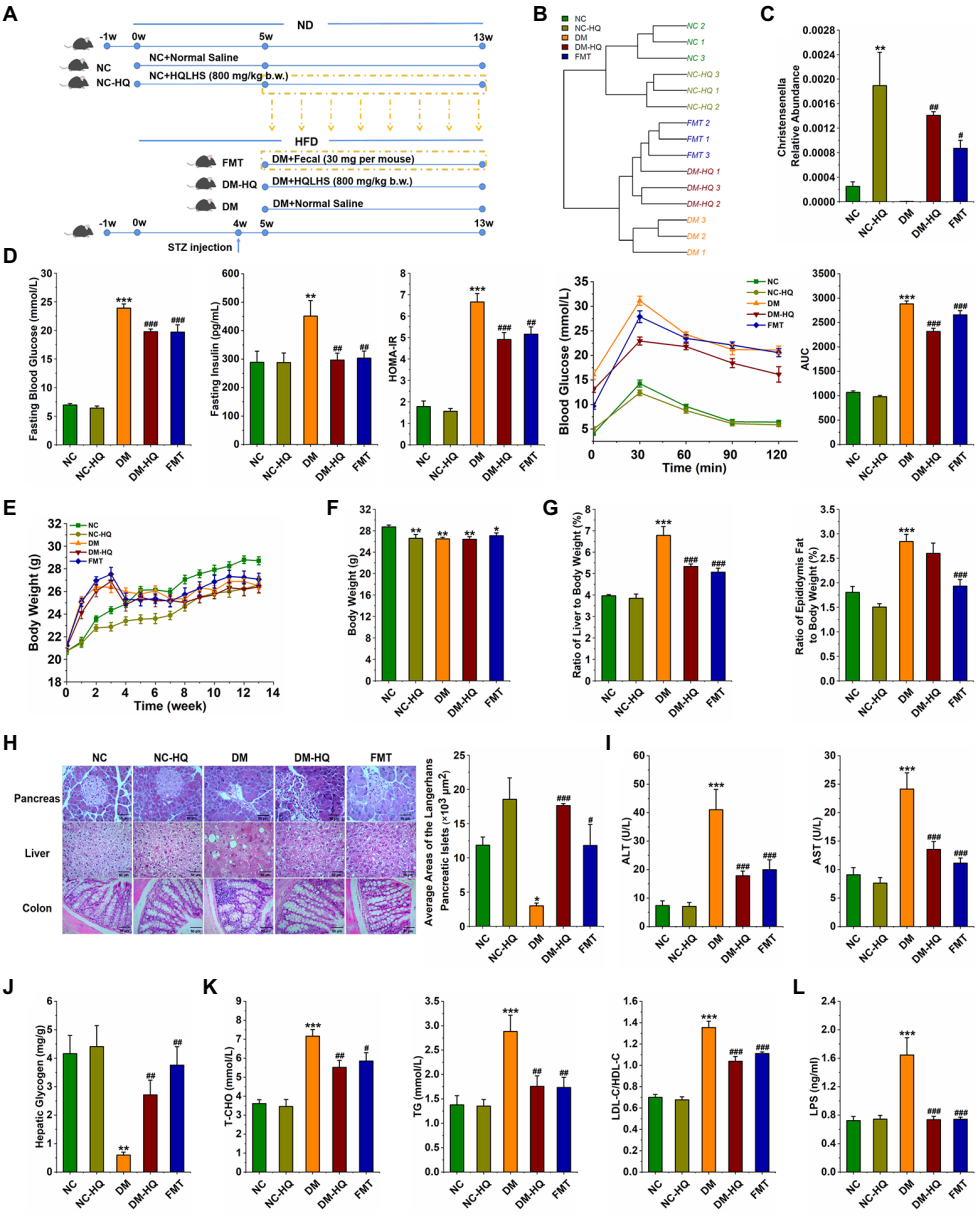
Treatment with fecal microbiota alleviated HFD and STZ induced type 2 diabetes. **(A)** Experimental design. **(B)** Hierarchical clustering tree of the cecum microbiota. **(C)** Relative abundance of *Christensenella* of five groups at week 13. **(D)** Fasting blood glucose, serum level of fasting insulin, HOMA-IR, OGTT test, and area under the curve (AUC). **(E)** The weekly weight changes. **(F)** Body weight at week 13. **(G)** The ratio of epididymis to body weight and the ratio of liver to body weight, respectively. **(H)** Morphological observation of pancreas (magnification, ×200, 50 μm), liver (magnification, ×200, 50 μm), colon (magnification, ×400, 50 μm) with H&E staining, and the average area of the pancreatic islets of Langerhans. **(I)** Serum ALT and AST levels. **(J)** Liver glycogen level. **(K)** Serum triglycerides, the total cholesterol, LDL-C/HDL-C levels. **(L)** Serum LPS level. Data are expressed as mean ± SEM; One-way ANOVA was used to analyze statistical differences; Compare to NC: *p < 0.05; **p < 0.01; and ***p < 0.001; Compare to DM: ^#^*p* < 0.05; ^##^*p* < 0.01; and ^###^*p* < 0.001.

After week 5, we collected feces of NC-HQ mice, and transplanted daily to the diabetic mice. The Part II experimental design is shown in Figure 4A. We analyzed the profile of cecal content microflora after the treatments according to 16S rDNA sequencing results. The hierarchical clustering tree shows close clustering pattern between the FMT and the NC-HQ group, suggesting successfully colonized most of the donor’s flora (Figure 4B). As in previous results, the α diversity of the five groups (Table 2) indicates that HQLHS did not alter the number of intestinal bacteria in diabetic mice.

**Table 2:** Effects of FMT on α diversity of cecal microbiota in diabetic mice.

	ACE	Chao1	Simpson	Shannon
NC	439.64 ± 16.41^b^	442.26 ± 18.24^b^	0.74 ± 0.04^c^	3.26 ± 0.23^c^
NC-HQ	438.11 ± 12.63^b^	448.00 ± 15.13^a^^,^^b^	0.86 ± 0.01^b^	4.02 ± 0.38^b^
DM	446.49 ± 3.21^a^^,^^b^	453.00 ± 2.79^a^^,^^b^	0.89 ± 0.04^b^	4.63 ± 0.45^a^^,^^b^
DM-HQ	448.08 ± 4.17^a^	455.09 ± 6.68^a^^,^^b^	0.90 ± 0.028^b^	4.9 ± 0.21^a^
FMT	465.40 ± 4.40^a^	467.87 ± 4.78^a^	0.93 ± 0.014^a^	5.13 ± 0.24^a^

Data were expressed as mean ± SEM.

a Means with different superscripts within the same row differ based on Tukey’s test (*p* < 0.05).

b Means with different superscripts within the same row differ based on Tukey’s test (*p* < 0.05).

c Means with different superscripts within the same row differ based on Tukey’s test (*p* < 0.05).

Principal co-ordinates analysis shows a clear difference in the flora structure among the five groups (Supplementary Figure 4 A). To be specific, there was no significant difference among the groups at the phylum level (Supplementary Figure 4 B). However, the abundances of *Lachnospiraceae* and *Ruminococcaceae* were significantly altered after the fecal microbiota transplantation (Supplementary Figures 4 C,D). At the genus level, the relative abundances of *Romboutsia* and *uncultured_bacterium_f_Muribaculaceae* were significantly raised after the fecal treatment, whereas the relative abundances of *uncultured_bacterium_f_Lachnospiraceae*, *Family_XIII_AD3011_group* and *Clostridium_sensu_stricto_1* were declined (Supplementary Figure 4 E). Interestingly, in the NC-HQ, DM-HQ, and FMT groups, *Christensenella* was considerably regulated upward (Figure 4C). Therefore, we speculated that HQLHS could preferentially enrich *Christensenella* taxa.

Importantly, feces transplantation from NC-HQ mice markedly attenuated T2D caused by HFD and STZ. In the FMT group, levels of fasting blood glucose, fasting insulin, HOMA-IR, and OGTT were reduced compared with the DM group (Figure 4D). Although body weight did not change, the weight indices of liver and epididymis fat were dramatically reduced in the FMT group (Figures 4E–G). Meanwhile, FMT reduced the serum levels of AST, ALT, TG, T-CHO, and LDL-C/HDL-C (Figures 4I,K). Fecal treatment also promoted liver glycogen synthesis (Figure 4J). FMT mice displayed recovery morphology of the liver, pancreas, and colon (Figure 4H). Moreover, FMT and HQLHS significantly reduced the content of LPS in serum (Figure 4L).

### Supplementation with *Christensenella minuta* or *Christensenella timonensis* improves T2D

While *Christensenella* is highly heritable and tied strongly to host health, its reduction is observed in obesity, Crohn’s disease, UC, and irritable bowel syndrome according to the previous reports (Waters and Ley, 2019; Li X, et al., 2020; Déjean et al., 2021). Based on the above results, *Christensenella* was notably enriched after HQLHS treatment, which negatively correlated with hyperglycemia and hyperlipemia. Thus, we speculated that *Christensenella* was the key bacterium in HQLHS treating T2D. To test this hypothesis and further understand the potential mechanism, diabetic mice were supplied with the strains *Christensenella minuta* DSM 22607 (CM) and *Christensenella timonensis* DSM 102800 (CT; part III design shown in Supplementary Figure 5 A).

**Figure 5:**
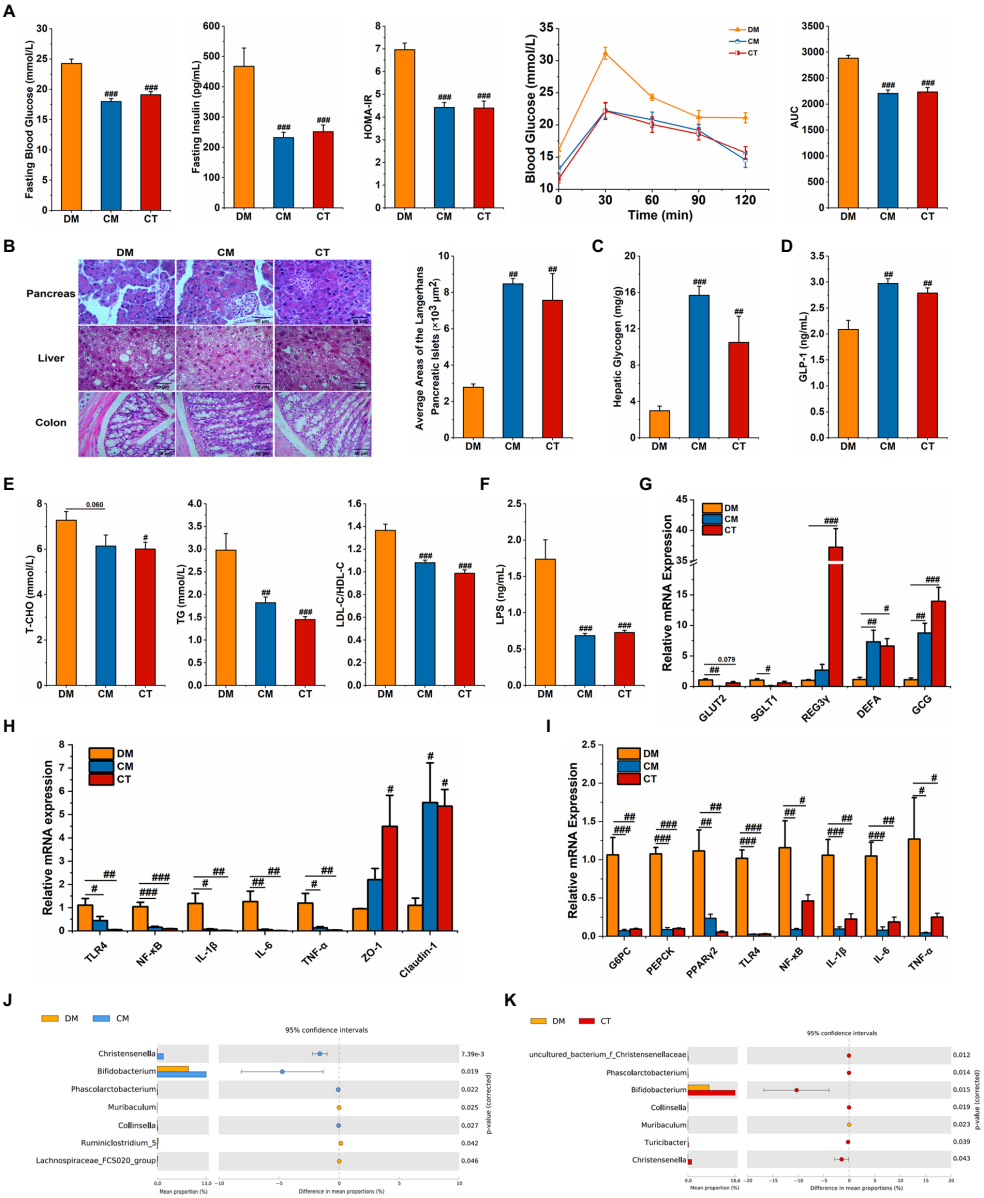
Supplementation with *Christensenella* ameliorated HFD and STZ induced type 2 diabetes. **(A)** Fasting blood glucose, serum level of insulin, homeostasis model assessment-insulin resistance (HOMA-IR), OGTT test, and area under the curve (AUC). **(B)** Morphological observation of pancreas (magnification, ×200, 50 μm), liver (magnification, ×200, 50 μm), colon (magnification, ×400, 50 μm) with H&E staining, and average areas of the pancreatic islets of Langerhans. **(C)** Liver glycogen level. **(D)** Serum GLP-1 level. **(E)** Serum triglycerides, cholesterol, LDL-C/HDL-C levels. **(F)** Serum LPS level. **(G)** Relative mRNA expression of GLUT2, SGLT1, and GCG in ileum tissue. **(H)** Relative mRNA expression of TLR4, NF-κB, IL-1β, IL-6, TNF-α, ZO-1, and Claudin-1 in colon tissue. **(I)** Relative mRNA expression of G-6-Pase, PEPCK, TNF-a, TLR4, NF-κB, IL-1β, and IL-6 in the liver tissue. **(J)** Differential abundant feature of genus between the DM and the CM group. **(K)** Differential abundant feature of genus between the DM and the CT group. Data are expressed as mean ± SEM; Significant differences between the two groups were analyzed using Student’s *t*-test; One-way ANOVA was used to analyze statistical differences; Compare to DM: ^#^*p* < 0.05; ^##^*p* < 0.01; ^###^*p* < 0.001.

Both strains of *Christensenella* could attenuate the abnormal blood glucose and lipid and restore the destruction of islet cells and the injury of the liver induced by STZ and HFD (Figures 5A–E; Supplementary Figures 5 B,D). Also, in CM and CT groups, less oxidative stress was observed, manifested by higher serum CAT, SOD and GSH-PX activity and lower serum MDA content (Supplementary Figure 5 C).

In both the treatment groups, the expression of colonic ZO-1 and Claudin-1 was regulated upwards (Figure 5H). In addition, LPS levels in serum decreased after *Christensenella* treatment (Figure 5F), and hepatic and colonic expressions of TLR4, NF-κB, IL-1β, IL-6, and TNF-α were suppressed (Figures 5H,I). The ELISA and qPCR examinations reveal that the CM and CT not only significantly stimulated the expression of proglucagon (GCG), the precursor gene encoding GLP-1, but also increased the serum GLP-1 content (Figures 5D,G). The CM and CT groups also reduced hepatic expression of two gluconeogenic rate-limiting enzymes, G6PC and PEPCK (Figure 5I). Besides, we found that only CM had notably inhibitory effect on the expressions of GLUT2 and SGLT1 in ileum (*p* < 0.05; Figure 5G). To sum up, *Christensenella* can improve glycolipid metabolism and reduce inflammatory response in diabetic mice.

Simultaneously, we monitored the cecal microflora in the three groups of mice; compared to the DM group, the relative content of *Christensenella* in the CM group and the CT group increased significantly (Figures 5J,K). In addition, the CM group showed significantly increased relative abundances of *Bifidobacterium*, *Phascolarctobacterium*, and *Collisella*, and a significant decrease the abundance of *Muribaculum*, *Ruminiclostridium_5* and *Lachnospiraceae_FCS020_group*. In the CT group, there was a significant increase in *uncultured_bacterium_Christensenellaceae*, *Bifidobacterium*, *Phascolarctobacterium*, and *Turicibacter*, and a significant decrease in the relative abundance of *Muribaculum*. Similar to the results of the Chinese medicine formula, the *Christensenella* supplementation also up-regulates the levels of beneficial bacteria.

**Figure 6:**
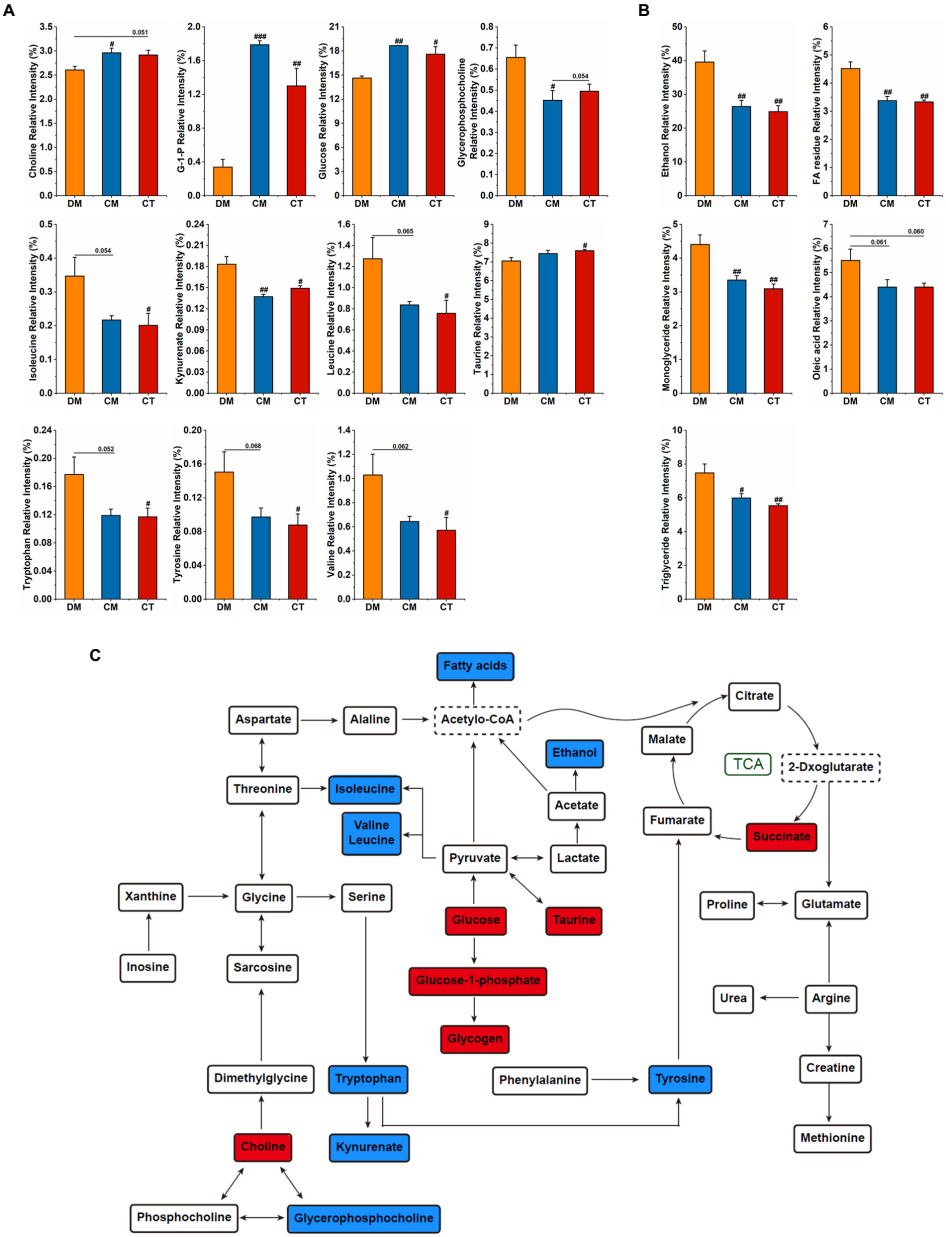
Supplementation with *Christensenella* affected liver metabolism. **(A)** Significant changes of relative intensity of hydrophilic metabolites. **(B)** Significant changes of relative intensity of lipophilic metabolites. **(C)** Main metabolite pathways of the liver of diabetic mice treated by *Christensenella*. The red/blue fillers indicate that the metabolite content increased/decreased after Christensenella treatment compared with the DM group.

To investigate the effect of these strains on hepatic metabolism, ^1^H NMR was performed to detect the hydrophilic and lipophilic metabolites, and the spectra are displayed in Supplementary Figure 6. OPLS-DA analysis presents a clear separation among the three groups (Supplementary Figures 7 A,B). The variables of VIP > 1 could be considered as potential biomarkers related to diabetes, among which the significantly changed metabolites were analyzed. In the CM and CT groups, we observed higher relative contents of choline, glucose-1-phosphate (G-1-P), glucose, taurine, and lower relative contents of valine, leucine, isoleucine, tryptophan, tyrosine, ethanol, FA residue, monoglycerides, oleic acid, and triglyceride (Figures 6A–C). The identified potential target metabolic pathways are shown in Figure 6C. Compared to the diabetic mice, MetaboAnalyst showed that CM mainly influenced Phenylalanine, tyrosine, and tryptophan biosynthesis; D-Glutamine and D-glutamate metabolism; and Alanine, aspartate, and glutamate metabolism, whereas CT mainly adjusted Phenylalanine, tyrosine, and tryptophan biosynthesis, Taurine and hypotaurine metabolism; and Alanine, aspartate, and glutamate metabolism (Supplementary Figures 7 C,D).

## Discussion

Studies have shown that TCM can alter the structure of intestinal flora, thereby improving the function of diseased organs or tissues (Zhang et al., 2019; Gong et al., 2020). Although the microbiome literature on T2D appears chaotic at first glance, accumulating evidence has supported that the gut flora makes a profound impact on metabolic diseases (Crommen and Simon, 2018; Fraga et al., 2019). Our research confirmed that HQLHS supplementation could reverse type 2 diabetes induced by HFD and STZ, which was accompanied by improvement in hyperglycemia, hyperlipemia, and oxidative stress. At the same time, we analyzed the regulation of HQLHS on intestinal microflora, among which the relative abundances of beneficial bacteria such as *Bifidobacterium*, *Romboutsia*, *Parabacteroides*, *Parvibacter*, and *uncultured_bacterium_f_Christensenellaceae* were raised. Similar structural changes in gut microflora have been reported, for instance, *Christensenellaceae* can be up-regulated by Okra extract in diabetic rats (Wu et al., 2021). Despite the emerging findings on the structure of gut microbiota, due to the factors of locality, individuality, and randomness, researchers have not yet reached an agreement on the composition of a healthy gut microbiome (Proctor, 2019). Thus, the contributions of specific microbes, their metabolic pathways, and their interactions with host health are the new focus of gut microbiota research.

Indeed, we show that HQLHS treatment led to accumulation of *Turicibacter*, *Alistipes*, *Bifidobacterium*, *Romboutsia*, *Christensenella*, and certain taxa of *Ruminococcaceae*, whether in diabetic or in normal mice. HQLHS treatment improves diabetic mice by modifying the intestinal flora. Similar studies have demonstrated that it is logical to use FMT in metabolic diseases to verify the causal relationship between oral drugs and intestinal flora (Gupta et al., 2016). By means of FMT, Wei et al. (2020) demonstrated that Sennoside A alleviates T2D through the regulation of intestinal flora. To enrich more beneficial bacteria, we chose the NC-HQ group as the FMT donor. The related indicators of hyperglycemia and hyperlipidemia were improved after FMT, and the relative abundances of *Turicibacter*, *Bifidobacterium*, *Romboustia*, *Christensenella*, and *uncultured_bacterium_f_Muribaculaceae* were increased to a certain degree. These results highlight the crucial role of the enriched bacteria in HQLHS relieving type 2 diabetes.

In a study of non-obese diabetic (NOD) mice, incomplete colonization of low-incidence T1D fecal flora did not change the potential incidence of diabetes, but the incidence was reduced when the key strain *Akkermansia muciniphila* was transferred. Thus, the success of FMT seems to depend on the transfer of the key species (Hänninen et al., 2018; Hanssen et al., 2021). Currently, there is a lack of research to delve deeper into the decisive key bacteria that can influence the effect of pharmacological interventions. Accordingly, we focused our attention on the enriched bacteria and hypothesized that these bacteria could act as “probiotics.” The above bacteria are associated with the improvement of glucose and lipid metabolism. For example, the colonization of *Turicibacter sanguinis* reduces host systemic triglyceride levels and inguinal adipocyte size (Fung et al., 2019; Wu et al., 2020). The bidirectional Mendelian randomization analyses indicated that higher relative abundance of *Alistipes* was associated with decreased blood triglyceride concentration (Liu et al., 2022). The abundance of *Romboutsia* is higher in normal rats than in diabetes rats. *Bifidobacterium* has long been used as a probiotic to improve metabolic diseases because of its possible role in improving the intestinal barrier and enhancing immunity (Hampe and Roth, 2017; Wen et al., 2021). *Ruminococcaceae* was able to ferment complex carbohydrates in the gut to produce formic acid and acetic acid, and some of its genera were usually negatively correlated with inflammation and HOMA-IR levels (Zeng et al., 2021); Notably, Goodrich et al. (2014) first identified *Christensenellaceae* as heritable, and the subsequent FMT experiments showed that amendment with *C. minuta* reduced weight gain in mice, compared to those that received unamended feces. Mazier et al. reported that *C. minuta* DSM33407 could prevent diet-induced obesity and regulated the related metabolic markers such as glucose and leptin (Mazier et al., 2021). Our study shows that *Christensenella* is not only enriched by HQLHS, but also increases significantly after FMT. Thus, we consider that the Chinese medicine formula may specifically regulate *Christensenella* to play a therapeutic role.

Therefore, we investigated the beneficial effects of *Christensenella* on HFD and STZ induced diabetic mice. The two strains, *C. minuta* DSM 22607 and *C. timonensis* DSM 102800, improved diabetes-related indicators. Oxidative stress plays an important role in the development of T2D (Mirmiranpour et al., 2013), and the continuous increase in blood glucose leads to the excessive production of ROS, resulting in tissue damage. Antioxidant enzymes, such as CAT, SOD, and GSH-PX, bind to and neutralize ROS in biological antioxidant systems (Bhatti et al., 2022). MDA is the final product of lipid peroxidation, and its content reflects the degree of tissue peroxidation damage (Song et al., 2022). Our results indicate that *Christensenella* reduces oxidative stress in diabetes by regulating the levels of antioxidant enzymes and MDA. Generally, the ability of intestinal bacteria to improve diabetes works through the intestinal tract, where enteroendocrine L-cells secrete GLP-1, encoded by the proglucagon gene (GCG), stimulating insulin secretion and regulating glucose homeostasis (Taşçı and Bingöl, 2018; Qin et al., 2021). The increased serum GLP-1 and GCG expression suggests that *Christensenella* intervention promotes GLP-1 secretion (Figures 5D,G). The Na^+^/glucose cotransporter SGLT1 triggers glucose uptake in the small intestinal epithelial mucosa, whereas GLUT2 promotes the entry of dietary sugars, glucose, fructose, and galactose into the bloodstream (Gavrilova et al., 2003). The role of SGLT1 and GLUT2 in intestinal glucose transport makes them potential targets for anti-diabetic therapy (Schmitt et al., 2017). *Christensenella minuta* DSM22607 significantly inhibited their expression suggesting an inhibition of glucose absorption. Moreover, recent evidence proves that lipopolysaccharide (LPS), inducing innate immune responses, plays an important role in metabolic disorders and may be a trigger for the onset of insulin resistance (Archer et al., 2021). The recognition of LPS by the receptor Toll-like receptor 4 (TLR4) leads to activation of nuclear factor kappa B (NF-κB) and expression of inflammatory regulatory genes, such as TNF-α, IL-1β, and IL-6 (Cani et al., 2008). Interestingly, *Christensenella* is Gram-negative, but we detected a decrease in serum LPS level. This may be due to the enhancement of the intestinal barrier characterized by reversed expression of ZO-1 and Claudin-1 and adjustment of intestinal flora composition. Yang et al. (2018) found that the structure of LPS of *C. minuta* is distinctly different from that of *Escherichia coli.* The former does not cause the proliferation, phagocytosis, and NF-κB activation of RAW 264.7 macrophages. In line with this finding, our results indicate that *Christensenella* administration reduces the inflammatory response induced by the LPS/TLR4/NF-κB pathway in diabetic mice. In general, *Christensenella* could control the blood glucose and blood lipids through multiple pathways.

The liver is the center of human metabolism, which not only maintains the balance of glucose and lipids, but also participates in the metabolism of amino acids and vitamins and many hormones (Brock and Dorman, 2007). In an NMR study of the effects of prebiotics and probiotics on liver health in obesogenic mice, *Bifidobacterium animalis* ssp. lactis 420 administration was found to significantly regulate the hepatic levels of betaine, taurine, and choline (Yde et al., 2021). In our work, *Christensenella* regulates mRNA expressions of G6PC and PEPCK in liver, levels of hepatic glycogen, and blood glucose and lipid. Therefore, we further explored the effects of *Christensenella* on liver metabolism in diabetic mice. Consistent with the serum indicators, supplement with the strains reduces fatty acid and triglyceride levels, and increases glucose and glycogen levels. BCAAs (leucine, valine, and isoleucine) and related metabolites have emerged as one of the strongest biomarkers for a range of cardiometabolic and related diseases, including obesity, insulin resistance, T2D, and coronary artery disease (Connelly et al., 2017; Yu et al., 2021). Moreover, in human metabolomics, plasma tryptophan levels and two kynurenine-pathway metabolites (kynurenate and xanthurenate) are associated with an increased risk of type 2 diabetes (Qi et al., 2021). In addition to lowering the levels of BCAAs, kynurenate, tryptophan, and tyrosine, *Christensenella* also increased taurine and succinate levels. Taurine can ameliorate oxidative stress in diabetic rats by regulating PI3K/Akt/GLUT4 pathway (Chen et al., 2021). Increased levels of taurine and succinate indicate an acceleration of the tricarboxylic acid cycle that enhances the oxidative metabolism. Meanwhile, the pathway analysis reveals that phenylalanine, tyrosine, and tryptophan biosynthesis is the pathway most affected by *Christensenella*. In general, the influence trend of the two strains on the body is consistent, notably affecting the metabolism of amino acids in the liver. Our study is the first to describe the effect of *Christensenella* on liver metabolites, laying the foundation for the understanding of its mechanism of action.

**Figure 7:**
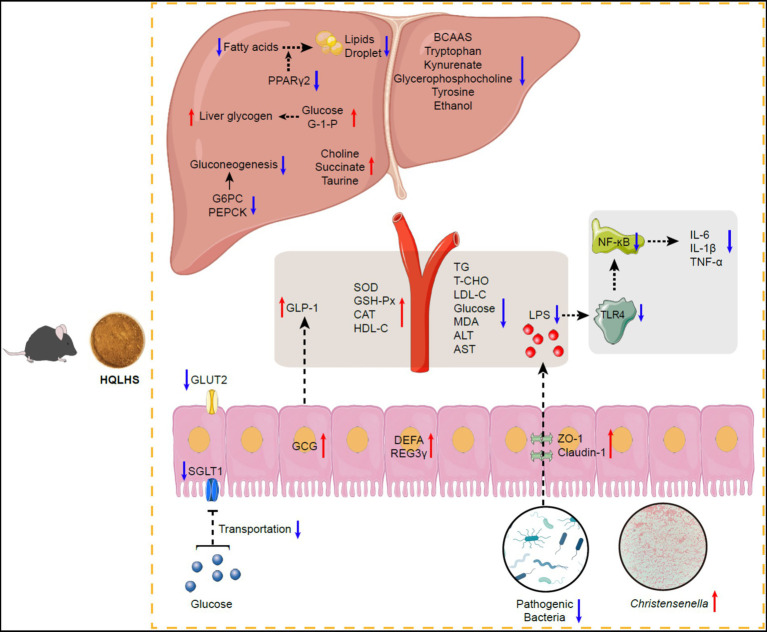
Schematic diagram showing the possible mechanisms of Huang-Qi-Ling-Hua-San ameliorating type 2 diabetes *via Christensenella*. HQLHS treatment inhibited harmful bacteria and enriched beneficial bacteria, especially *Christensenella*. *Christensenella* promoted the production of GLP-1; Reduced the expression of GLUT2 and SGLT1, thereby inhibiting the transport of glucose in the small intestine; Increased the expression of ZO-1 and Claudin-1, thus decreasing the entry of LPS into the periphery, and reduced inflammation through LPS/TLR4/NF-κB; Improved the activity of antioxidant enzymes; Decreased the expression of G-6-Pase and PEPCK, which inhibited gluconeogenesis; Increased the synthesis of liver glycogen; Regulated liver metabolites such as BCAAs, tryptophan, kynurenate, and taurine.

The proposed mechanism by which HQLHS plays a therapeutic role by regulating *Christensenella* is shown in Figure 7. Administration of HQLHS inhibits harmful bacteria and enriches beneficial ones, especially *Christensenella*, which alleviates type 2 diabetes by promoting GLP-1 secretion, regulating hepatic glucose metabolism, inhibiting intestinal glucose absorption, enhancing intestinal barrier, reducing inflammation *via* LPS/TLR4/NF-κB pathway, and improving liver metabolism. In summary, we indicate that Huang-Qi-Ling-Hua-San alleviates type 2 diabetes by enriching *Christensenella* taxa. This provides new insights into the search for key intestinal bacteria that respond to oral traditional Chinese medicine.

## Data availability statement

The datasets presented in this study can be found in online repositories. The names of the repository/repositories and accession number(s) can be found at: http://doi.org/10.57760/sciencedb.01693.

## Ethics statement

The animal study was reviewed and approved by Experimental Animal Welfare and Ethics Committee of School of Life Sciences, Jilin University.

## Author contributions

TP: conceptualization, investigation, visualization, and writing—original draft preparation. SZ, WZ, CS, KN, and QZ: investigation. YX: resources and supervision. HX and QX: conceptualization, resources, supervision, and writing—review and editing. All authors contributed to the article and approved the submitted version.

## Funding

This work was supported by the Department of Science and Technology of Jilin Province under (grant nos. 20200708072YY and 20210401122YY).

## Conflict of interest

The authors declare that the research was conducted in the absence of any commercial or financial relationships that could be construed as a potential conflict of interest.

## Publisher’s note

All claims expressed in this article are solely those of the authors and do not necessarily represent those of their affiliated organizations, or those of the publisher, the editors and the reviewers. Any product that may be evaluated in this article, or claim that may be made by its manufacturer, is not guaranteed or endorsed by the publisher.

## Footnotes

^1^ http://www.kegg.jp

^2^ http://www.hmdb.ca
