# Supplementary material for: Christensenella regulated by Huang-Qi-Ling-Hua-San is a key factor by which to improve type 2 diabetes
Source: Front Microbiol. 2022 Oct 12;13:1022403. doi: 10.3389/fmicb.2022.1022403 (PMC9597676; doi:10.3389/fmicb.2022.1022403)
Supplement: Supplementary file 1 [file Data_Sheet_1.DOCX]

**Figures**

**
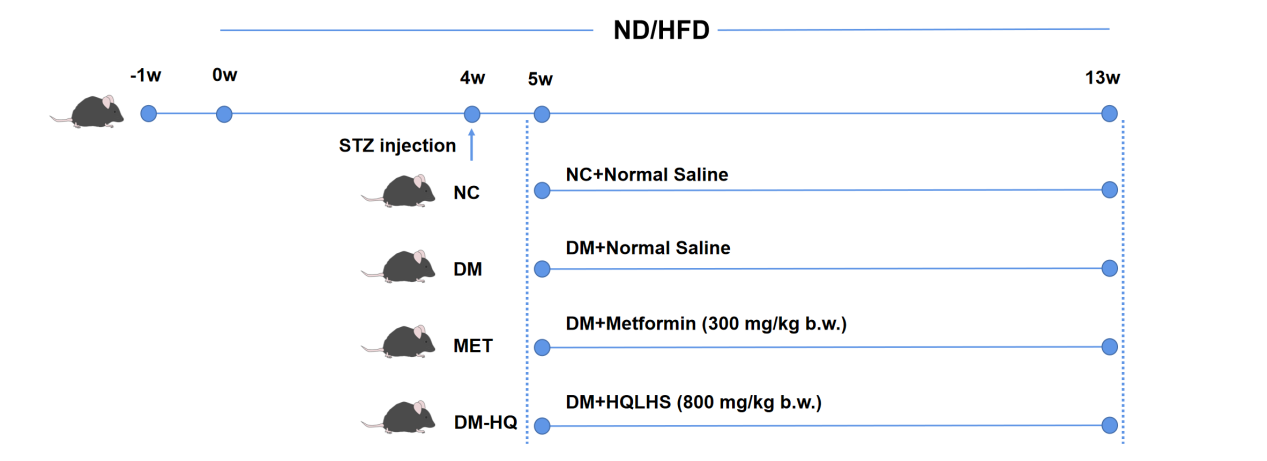
**

**Supplementary Figure 1.** Experimental design of part Ⅰ. After one week of acclimation, the male C57BL/6J mice were randomly divided into 4 groups (n=10) concluding 10 mice in the normal control (NC) group fed with normal diet and the remaining 30 mice fed with 60% high-fat diet, which were intraperitoneally injected with STZ (120 mg/kg) by the fourth week. Meanwhile, the NC group was injected with citrate buffer. From week 5: DM group mice received normal saline daily; MET group mice received (300 mg/kg/d) metformin; DM-HQ group mice received doses of HQLHS dissolved in normal saline by gavage (800 mg/kg/d).


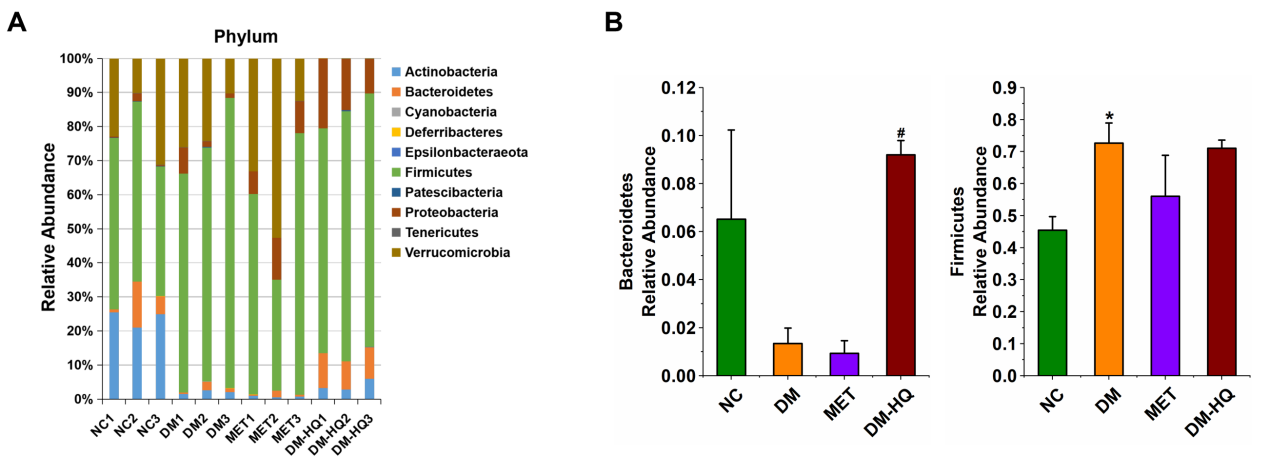


**Supplementary Figure 2.** (A) Relative abundance of cecal bacterial taxa at the phylum level. (B) Comparison of the relative abundance of Bacteroides and Firmicutes. Data are expressed as mean ± s.e.m.; One-way ANOVA was used to analyse statistical differences; Compare to NC: ******P*<0.05; *******P*<0.01; ********P*<0.001; Compare to DM: **#***P*<0.05; **##***P*<0.01; **###***P*<0.001.


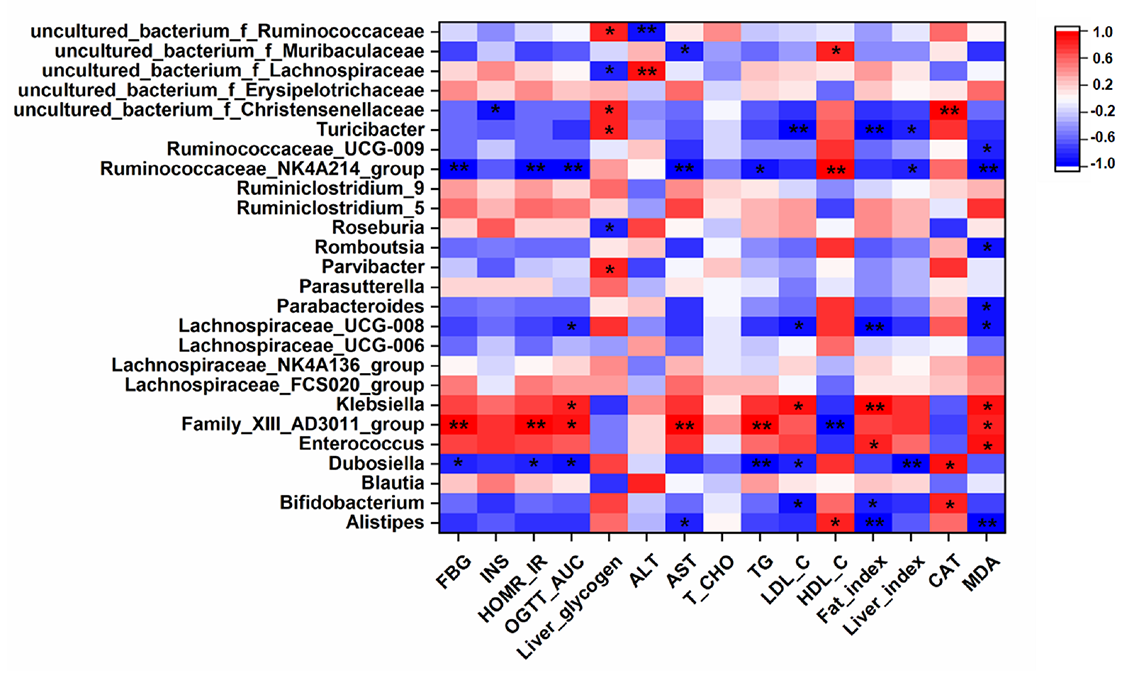


**Supplementary Figure 3.** Correlation analysis between cecal microbiota (genus level) reversed by HQLHS intervention with T2D related parameters based on Spearman’s rank correlation coefficient. The heat map represents the correlation coefficient value. Significant correlations are marked by ******P*<0.05, *******P*< 0.01.


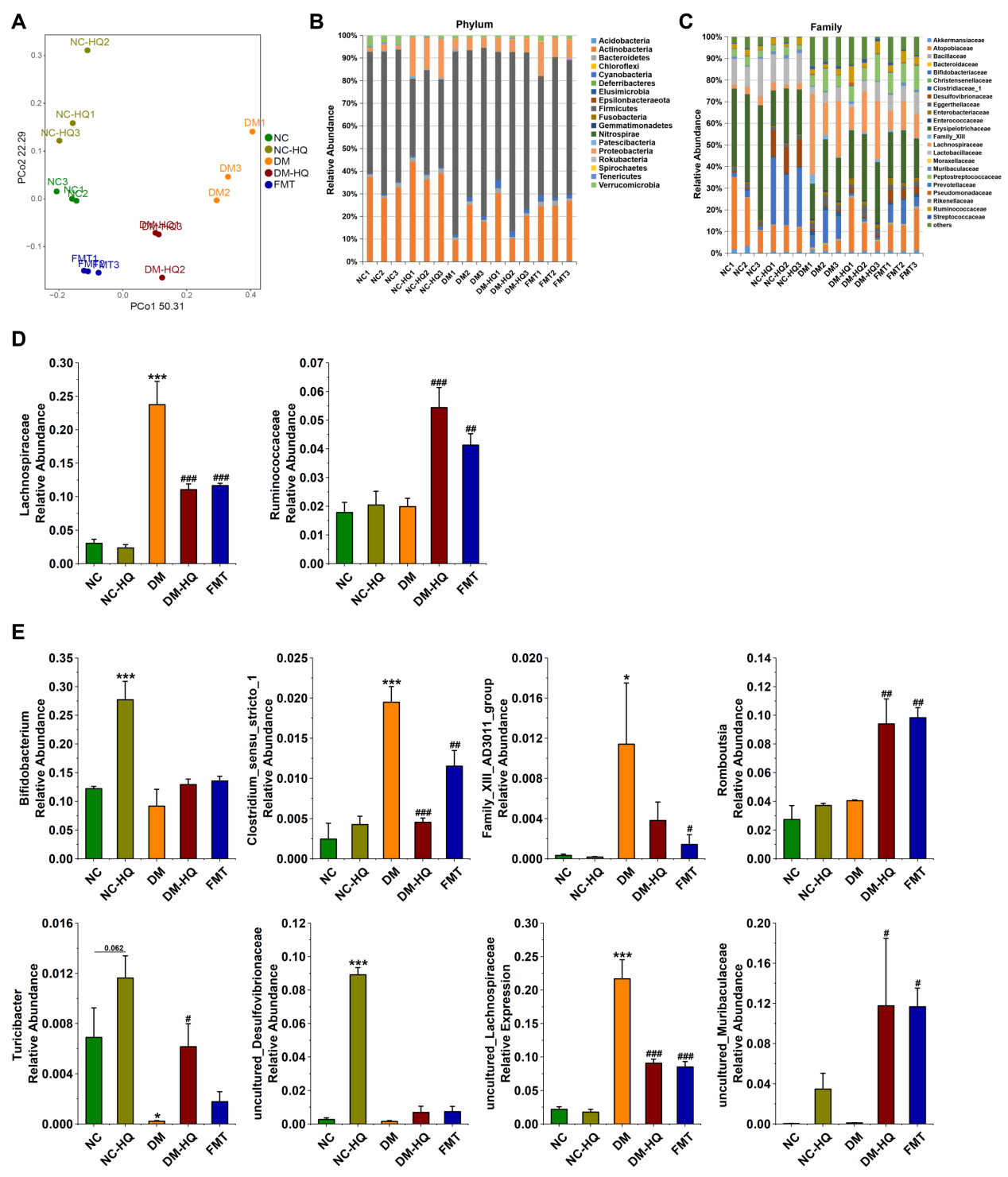


**Supplementary Figure 4.** (A) Principal co-ordinates analysis (PCoA) of cecum microbiota. (B) Phylum-level distribution of cecum microbiota. (C) Family-level distribution of cecum microbiota. Relative abundance of significantly altered bacterial taxa at (D) family and (E) genus levels among the five groups of mice. Data are expressed as mean ± s.e.m.; One-way ANOVA was used to analyse statistical differences; Compare to NC: ******P*<0.05; *******P*<0.01; ********P*<0.001; Compare to DM: **#***P*<0.05; **##***P*<0.01; **###***P*<0.001.


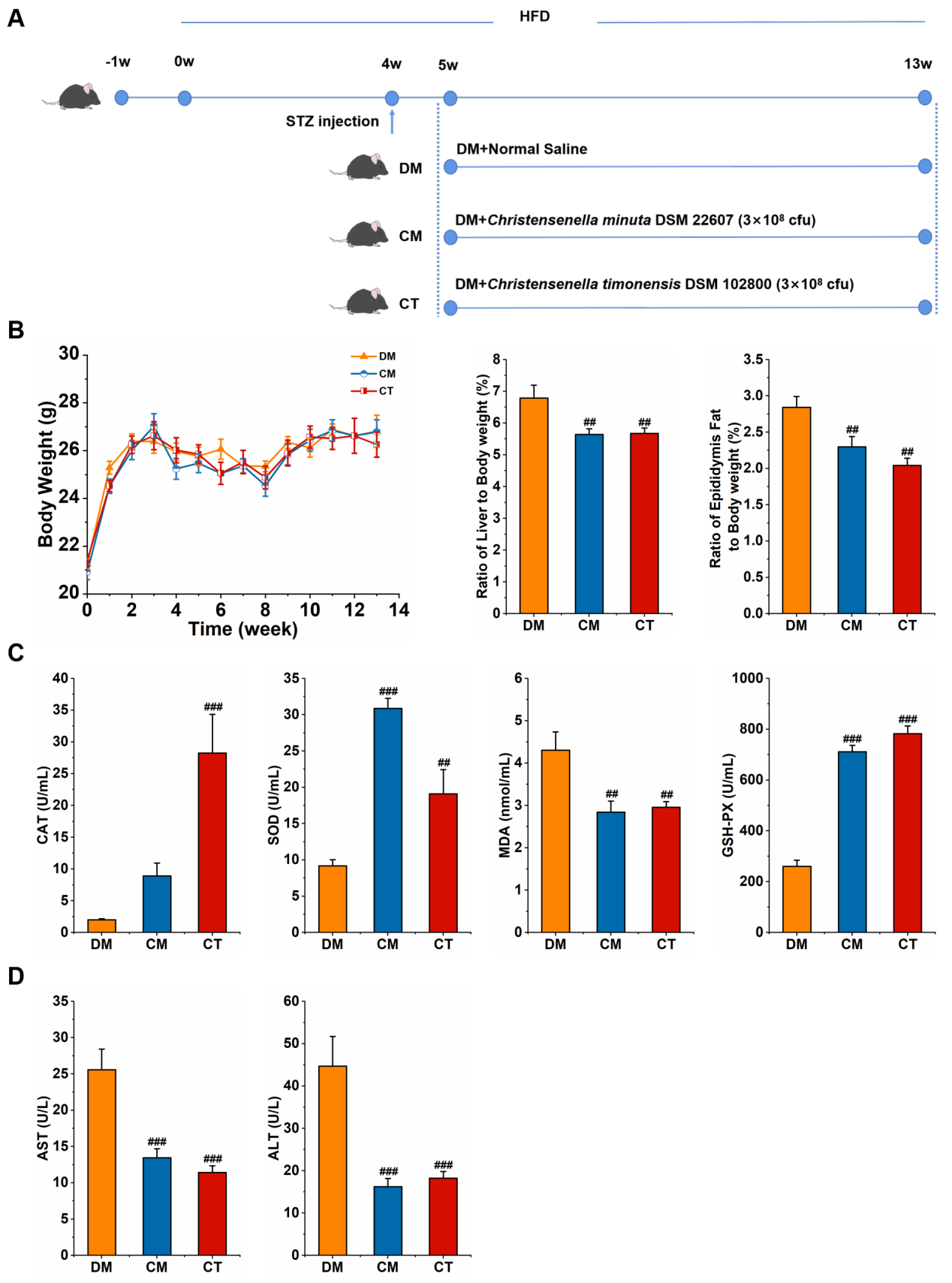


**Supplementary Figure 5.** (A) Part Ⅲ experimental design. (B) The weekly weight changes, the ratio of epididymis to body weight and the ratio of liver to body weight, respectively. (C) Serum levels of CAT, SOD, MDA and GSH-PX. (D) Serum levels of AST and ALT. Data are expressed as mean ± s.e.m.; One-way ANOVA was used to analyse statistical differences; Compare to DM: **#***P*<0.05; **##***P*<0.01; **###***P*<0.001.


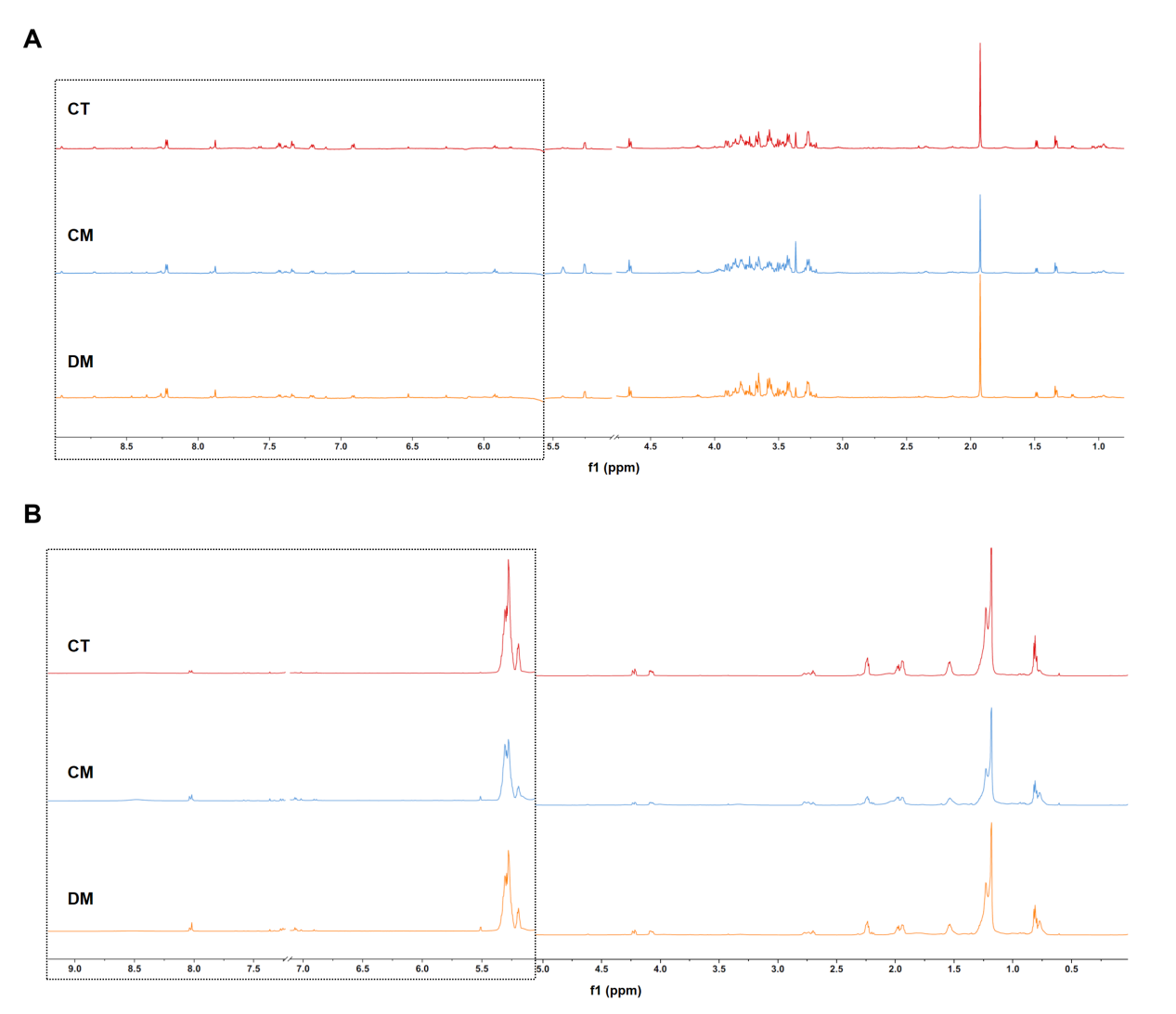


**Supplementary Figure 6.** (A) 600 MHz ^1^H NMR spectrum of aqueous extract from mice liver. In order to better display the spectrum, part δ5.5-10.0 (dotted box) of the spectrum is 15 times larger than δ1.0-5.5 in the vertical direction. (B) 600 MHz ^1^H NMR spectrum of lipid extract from mice liver. In order to better display the spectrum, part δ5.0-9,5 (dotted box) of the spectrum is 15 times larger than δ0.0-5.0 in the vertical direction.


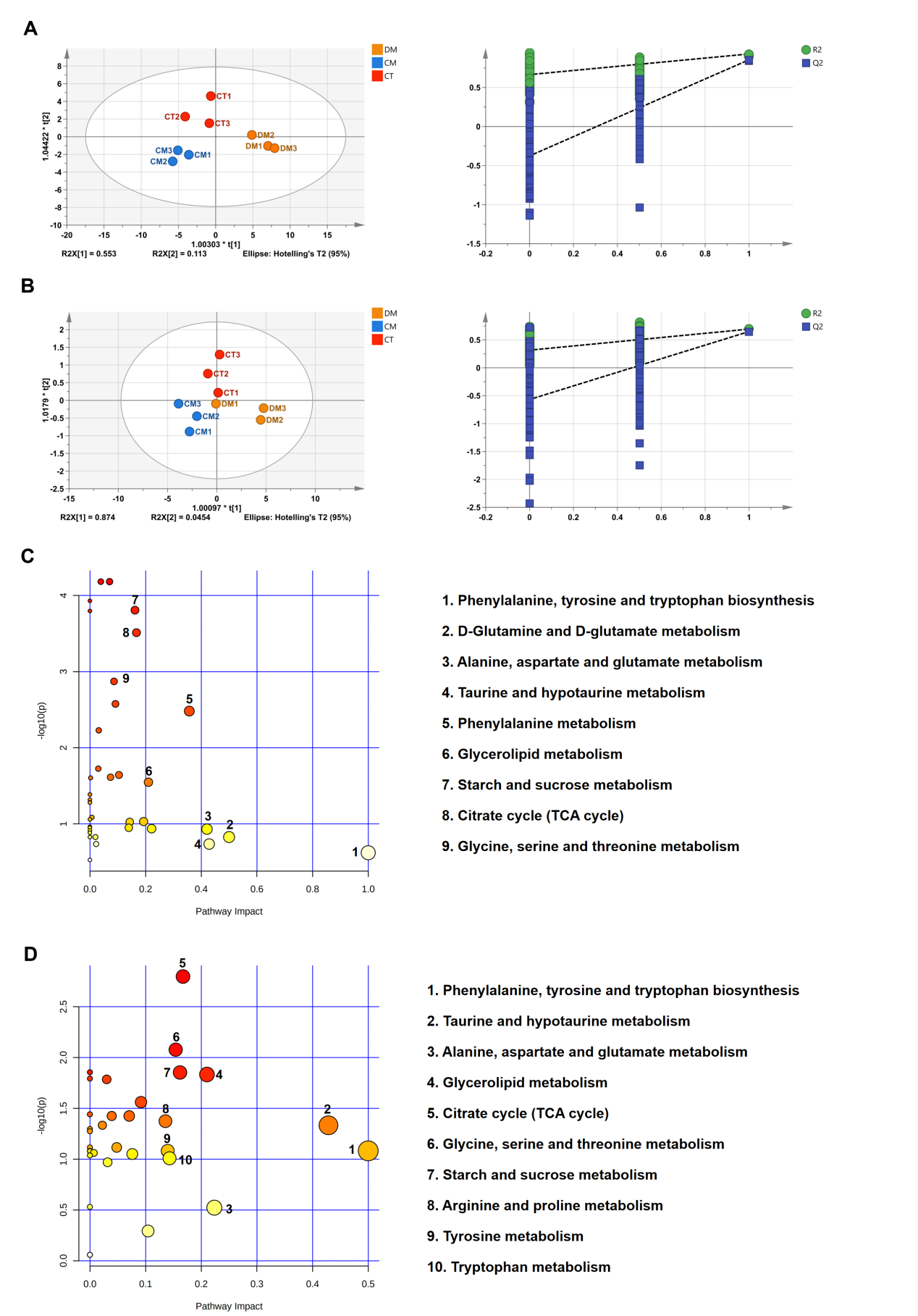


**Supplementary Figure 7.** (A) Liver hydrophilic metabolites of OPLS-DA score plots and the corresponding validation plots based on 200 times permutation tests. (B) Liver lipophilic metabolism of OPLS-DA score plots and the corresponding validation plots based on 200 times permutation tests. (C) Analysis of the liver metabolic pathway after *C. minuta* DSM 22607 treatment. (D) Analysis of the liver metabolic pathway after *C. timonensis* DSM 102800 treatment.
