# Supplementary material for: Christensenella regulated by Huang-Qi-Ling-Hua-San is a key factor by which to improve type 2 diabetes
Source: Front Microbiol. 2022 Oct 12;13:1022403. doi: 10.3389/fmicb.2022.1022403 (PMC9597676; doi:10.3389/fmicb.2022.1022403)
Supplement: Supplementary file 3 [file Table_2.DOCX]

**Supplementary Table 2.** Quantitative Real-time PCR primer sequences

| Gene | Sequence(5' to 3') | Sequence(5' to 3') |
| --- | --- | --- |
| 18S | AGGCGCGCAAATTACCCAATCC | GCCCTCCAATTGTTCCTCGTTAAG |
| GLUT2 | TCAGAAGACAAGATCACCGGA | GCTGGTGTGACTGTAAGTGGG |
| G-6-pase | AGGAAGGATGGAGGAAGGAA | TGGAACCAGATGGGAAAGAG |
| PEPCK | TGACAGACTCGCCCTATGTG | CTGCATAACGGTCTGGACTTC |
| GCG | TGGTTGGAACCTTGGTGAATA | GCTGCAGCCCATTAAGATG |
| SGLT1 | TACCTGAGGAAGCGGTTTGGA | CGAGAAGATGTCTGCCGAGA |
| TLR4 | CCAGAGCCGTTGGTGTATCT | GGCGATACAATTCGACCTGC |
| NF-κB | ATGGCAGACGATGATCCCTAC | TGTTGACAGTGGTATTTCTGGTG |
| IL-6 | TCCAATGCTCTCCTAACAGATAAG | CAAGATGAATTGGATGGTCTTG |
| IL-1β | TCCATGAGCTTTGTACAAGGA | AGCCCATACTTTAGGAAGACA |
| TNF-α | CGAGTGACAAGCCTGTAGCC | CATGCCGTTGGCCAGGA |
| ZO-1 | TTTTTGACAGGGGGAGTGG | TGCTGCAGAGGTCAAAGTTCAAG |
| Claudin-1 | CGGGCAGATACAGTGCAAAG | ACTTCATGCCAATGGTGGAC |
