# Supplementary material for: Christensenella regulated by Huang-Qi-Ling-Hua-San is a key factor by which to improve type 2 diabetes
Source: Front Microbiol. 2022 Oct 12;13:1022403. doi: 10.3389/fmicb.2022.1022403 (PMC9597676; doi:10.3389/fmicb.2022.1022403)
Supplement: Supplementary file 4 [file Table_3.DOCX]

**Supplementary Table 3.** ^1^H NMR spectrogram attribution table of liver metabolites

| **Hydrophilic/Lipophilic** | **chemical shif (ppm)** | **Assignment** |
| --- | --- | --- |
| Pantothenate | 0.94(s) | CH_3_ |
| Leucine | 0.96(t) | CH_3_; CH_2_&γ-CH |
| Valine | 0.99(d); 1.04(d) | γ-CH_3_; γ-CH’_3_ |
| Isoleucine | 1.01(d) | β-CH_3_ |
| Ethanol | 1.19(t); 3.63(q) | CH_3_; CH_2_ |
| LDL | 1.21(s) | (CH_2_)n |
| Lactate | 1.32(d)；4.13(q) | β-CH_3_; CH |
| Alanine | 1.49(d) | CH_3_ |
| Arginine | 1.73(m) | β-CH_3_ |
| Acetate | 1.92(s) | CH_3_ |
| Proline | 2.0(m) | γ-CH_2_ |
| Methionine | 2.14(s); 2.16(m) | δ-CH_3_; CH_2_ |
| Glutamate | 2.33(m) | γ-CH_2_ |
| Succinate | 2.41(s) | CH |
| Acetylcarnitine | 2.51(m); 2.63(m) | CH_2_; β-CH_2_ |
| Citrate | 2.55(d) | CH_2_(1/2) |
| Aspartate | 2.69(m) | β-CH |
| Asparigine | 2.84(dd) | β-CH |
| Creatine | 3.03(s) | CH_3_ |
| Phosphocholine | 3.21(s); 3.59(m) | N(CH_3_)_3;_ N-CH_2_ |
| Glycerophosphocholine | 3.23(s) | CH_3_ |
| TMAO | 3.26(s) | N-(CH_3_)_3_ |
| Taurine | 3.27(t); 3.43(t) | CH_2_SO_3_; NCH_2_ |
| Phenylalanine | 3.28(m); 7.33(d); 7.38(t); 7.43(t) | β-CH’; o-CH; p-CH; m-CH |
| Methanol | 3.37(s) | CH_3_ |
| β-Glucose | 3.41(t); 3.46(m); 3.90(dd) | CH(4); CH(5); CH(6’) |
| Choline | 3.50(t/m) | N-CH_2_ |
| Glycine | 3.58(s) | N-CH_2_ |
| Malate | 3.57(dd) | CH’(2) |
| Sarcosine | 3.59(s) | CH_2_ |
| N,N-Dimethylglycine | 3.72(s) | CH_3_ |
| α-Glucose | 3.74(m); 3.84(m); | CH(6); CH(5&6’) |
| Glutathione | 3.78(m) | α-CH&CH, -NH |
| Hippurate | 3.96(d); 7.54(t) | C_3_H, ring |
| Threonine | 4.26(m) | β-CH |
| Inosine | 4.28(dd); 4.44(dd); 8.35(s) | CH(5); CH(4); N-CH’=N |
| Trehalose | 5.19(d) | CH(2, 8) |
| Maltose | 4.66(d); 5.24(d) | CH(1); CH’(1) |
| Glucose-1-phosphate | 5.42(q) | 1-CH |
| Uracil | 5.81(d) | CH(5) |
| Uridine | 5.91(d) | CH(2) |
| Fumarate | 6.52(s) | CH |
| Tyrosine | 6.91(d) | m-CH |
| Histidine | 7.08(s) | CH(5) |
| Tryptophan | 7.20(t) | C_4_H, ring |
| Phenylacetyl glycine | 7.42(t) | 3,5-CH |
| Nicotinic Acid | 7.61(dd) | CH(5) |
| 4-Aminohippurate | 7.73(d) | CH |
| Uridine | 7.87(d) | CH(11) |
| Kynurenate | 7.88(d) | 6-CH(ring) |
| 4-Pyridoxate | 7.91(s) | CH(3) |
| Hypoxanthine | 8.20(s); 8.21(s) | CH(2); CH(7) |
| Formate | 8.46(s) | CH |
| Nicotinamide | 8.72(dd) | CH(6) |
| 1-Methylnicotinamide | 8.96(d) | 2-CH |
| Cholesterol | 0.66(s) | C_18_H_3_ |
| FA residue | 1.53(m) | CH_2_ |
| FA, Oleic acid | 2.00(m) | CH_2_ |
| Monoglycerides | 2.23(m) | CH_2_ |
| FA, PUFA | 2.80(m) | CH_2_ |
| Triglycerides | 3.89(m); 4.22(dd); 5.26(m) | C_1_H; C_3_H |
| Unknown | 4.61(s); 8.02(d) | - |

s:single peak;d: double peaks; t: triple peaks; q: quadruple peaks; dd: double double peaks; m: multiple peaks.
